# Supplementary material for: X-chromosome and kidney function: evidence from a multi-trait genetic analysis of 908,697 individuals reveals sex-specific and sex-differential findings in genes regulated by androgen response elements
Source: Nat Commun. 2024 Jan 18;15:586. doi: 10.1038/s41467-024-44709-1 (PMC10794254; doi:10.1038/s41467-024-44709-1)
Supplement: Supplementary file 1 — Supplementary Information [file 41467_2024_44709_MOESM1_ESM.pdf]

X-chromosome and kidney function: Evidence from a multi-trait genetic analysis of 908,697 individuals reveals sex-specific and sex-differential findings in genes regulated by androgen-response elements

Table of Contents

Supplementary Notes 1

Supplementary Note 1 1

Supplementary Note 2 6

Supplementary Figures 14

Supplementary References 34

Supplementary Notes

Supplementary Note 1

In the following, we provide detailed insights into the different locus findings.

Locus 1 (Xp22.31): Strongest association was observed for the variant rs139036121 for eGFR in males. BUN was not significant. We observed a pronounced sex-interaction and no association in females. Thus, this association is male-specific (**Figure 3**). The variant was nominally significantly associated with CKD, UACR and MA but not BUN. The variant is in LD with variants reported by Graham et al., Kanai et al. and Sakaue et al.[1–3], but here, we demonstrated that the hit is male-specific. The locus is pleiotropic with a variety of other GWAS associations including other sex-specific traits such as testosterone and male-pattern baldness[4,5]. Moreover, our eGFR signal colocalizes with a signal of testosterone in males (PP(H4)=99%) with opposite effect directions, i.e. the eGFR signal could be driven by a primary testosterone effect (**Supplementary Data 14**).

The credible set contained 17 variants, none of them with a pronounced CADD score. No eQTLs of relevant tissue were in LD and no colocalizations with eQTLs were observed for this locus. The nearest candidate is *FAM9B*, which has no obvious link to kidney function. Of note, *FAM9B* has an androgen response element (ARE) upstream to transcription start side (TSS) (70kB)[6].

Locus 2 (Xp22.13): Strongest association was observed for rs5909184 for eGFR in the overall analysis without sex-interaction. The variant is also associated with BUN with opposite effect direction, i.e. kidney function is likely. The variant was also found in Graham et al. and Sakaue et al.[1,3] The credible set contains four variants, where the top-variant already accounts for 90% posterior probability. The variant is in the coding region of *CDKL5*. Moreover, a cis-eQTL signal of *CDKL5* in tubulointerstitial tissues of the kidney co-localizes with the eGFR signal with opposite effect direction (**Figure 5**). Thus *CDKL5* is a plausible candidate gene, which is associated with Rett syndrome with possible implications to kidney disease[7].

Locus 3 (Xp11.23): Strongest association was observed for rs72616719 for eGFR in the overall analysis without sex-interaction. The variant is also associated with BUN, i.e. its functional relevance to kidney is likely. The association was previously described in Sakaue et al.[3] The credible set contains 67 SNPs. The top-associated variant has the highest PP and also a high CADD score of 12.5. It is a gene-rich region. Colocalization with an eQTL of *NDUFB11* in muscle-skeletal tissue was observed as well as with *USP11* and *CDK16* in other tissues (**Figure 5, Supplementary Data 8**). The eQTLs of the latter two showed opposite effect direction compared to eGFR. Of note, *CDK16* is associated with renal cell carcinoma[8], while *USP11* is related to renal tubular cell senescence and fibrosis[9]. Thus, both genes are plausible candidates.

Locus 4 / 16 (Xq12): The index variant rs189618857 of this locus was associated with eGFR in males only with a strong sex-interaction effect (p_{IA}=1.5x10^{-3}, FDR<5%, **Figure 3**). BUN was not significant. The variant was not associated with BUN but with CKD and UA, again associations with these traits were not present (CKD) or much weaker (UA) in females compared to males. The locus overlaps with locus 16 of UA association (top-variant rs6625094), although this is due to LD but not colocalization (**Table 2**).

The locus was described for association with serum creatinine and uric acid levels by Sakaue et al.[3], but here, we demonstrate that it is male-specific. Other GWAS traits associated at this locus comprise among others sex hormone-binding globulin levels, male-pattern baldness, fasting insulin, estradiol levels with same effect direction and prostate cancer.The top-SNP rs189618857 is in a gene-desert but shows scattered support. The credible set of eGFR association comprised 537 variants with strong CADD score variants near *EDA2R*, which is a plausible candidate gene[10]. Co-localization was detected only for an eQTL of *OPHN1* in skin. However, LD with eQTLs of *EDA2R* and *AR* were observed for this locus (**Supplementary Data 7**). Since *AR* has upstream estrogene response elements[11], this is another plausible candidate gene of this locus[12]. According to Wilson et al., there is also an androgen response element 5kB upstream of the TSS[6]. *EDA2R* also has an ARE in some distance from the gene-body. Both genes were shown to be regulated by the ARE (*AR* up-regulated, *EDA2R* down-regulated[6]). Moreover, *AR* shows significantly higher gene-expression in females while *EDA2R* shows higher expression in males in several tissues[13]. Thus, we consider both, *EDA2R* and *AR* as plausible candidates here.

Locus 5 (Xq21.1): The locus was associated best in eGFR overall (rs2063579) without sex interactions. It was not associated with BUN but with CKD. The locus was also reported by Sakaue et al.[3] The credible set comprised 233 variants, while the index variant showed a strong deleteriousness estimate (CADD=17) and is in proximity to *BRWD3*. No eQTL colocalizations were observed. The functional relationship of this gene with kidney traits needs to be elucidated.

Locus 6 (Xq22.1): The locus was best associated with eGFR overall (rs1802288) without sex interactions. BUN was not associated but CKD and UA. The association with UA becomes significant after adjusting for ancestry with MR-MEGA (**Supplementary Figure 6, B**). No GWAS trait associations were described for that locus. The credible set contained only the index variant with a pronounced CADD score of 29.9. The SNP is a miss-sense mutation of *TSPAN6* (Ala108Thr). No eQTL colocalizations were detected. A relationship of this gene with kidney function was not yet described. However, of note, another member of the tetraspanin family, namely *TSPAN33* located at chromosome 7 was proposed as a candidate gene of eGFR association in the study of Graham et al.[1]

Locus 7 / 18 (Xq22.1): Highest association was observed for rs3850318 for eGFR in the overall analysis. The variant was also associated with BUN, CKD and UA, i.e. this association overlaps with locus 18 of UA (rs34884874, colocalization PP(H4)=93%). This association was in LD with associations of creatinine and UA as reported in Sakaue et al.[3] The credible sets of the top-hits comprised 126 variants. There is a high-CADD variant within *ARMCX4* (CADD=12). We observed eQTL colocalizations of the eGFR

respectively UA signals with *ARMCX2* in kidney tubulointerstitial tissue with opposite respectively same effect direction (PP(H4)=82% respectively 95%, **Figure 5**) prioritizing this gene.

Since colocalization analysis between male and female eGFR results at this locus strongly supported the hypothesis of different signals (PP(H3)=95%, **Supplementary Data 4**), we analyzed this phenomenon in more detail by looking at the sex-stratified results of eGFR. The top-variant in males was rs2858167, which is 62kB away from rs3850318, still the variants are in LD (r^2=0.83, **Figure 2**). The SNP did not achieve genome-wide significance in males and no significant sex-interaction was observed (p_{IA}=0.32). Conversely, the top-variant in females was rs149995096, which is 460kb away from rs3850318 and is not in LD with this variant nor the male top-hit (r^2<0.018). Of note, this variant achieved genome-wide significance in females while the effect in males was not even nominally significant (p_{IA}=5.1x10^{-4}). Thus, we consider this variant an independent female-specific hit of this locus. Moreover, this variant was not in LD with other reported GWAS, thus, representing a novel finding. CKD but not BUN was associated with this variant. The variant is in the coding sequence of *DRP2* and the credible set comprising 92 variants also contains high CADD score variants of this gene. *DRP2* could be a plausible candidate due to its relationship to creatinine via involvement in muscle dystrophy[14]. Since BUN is not significant, this association could be related to muscle mass. Of note, the gene has an ARE 17kb downstream of the TSS[6] and shows higher expression in females in several tissues[13]. Since there is no evidence of X-inactivation escape of this gene[15,16], this gene-expression difference is likely caused by different regulation but it is unlikely that this explains the observed eGFR association due to lack of colocalization of gene-expression and eGFR signals at this locus (**Supplementary Data 8**).

Locus 8/19 (Xq22.2): The locus is best associated for eGFR (overall analysis, rs11092455) without sex-interactions. BUN, CKD and UA were also associated. A physical overlap with locus 19 of UA (overall analysis) association (index variant rs34815154) was unclear based on both LD (r^2=0.28) and colocalization analyses (pp(H3)=45%, pp(H4)=54%). However, both signals are also colocalized with eQTLs of *TCEAL3* and *MORF4L2* in muscle-skeletal, whole blood with the same effect direction and other tissues as well (**Figure 5, Supplementary Data 8**). Moreover, index variants of both loci are in LD with a variant reported for UA association in Sakaue et al.[3] such that a locus overlap was considered likely. The credible set of eGFR associations contained 67 variants, while the index variant already carried 63% PP. The nearest gene is *MORF4L2* but there are also high CADD score variants near *TCEAL3*. Combined with the colocalization findings, we consider both genes as plausible candidates but their role in kidney function is unclear so far.

Locus 9 (Xq22.3): At this locus, the top variant is rs181497961 showing highest association with eGFR in the overall analysis and no sex-interaction. The variant is also associated with BUN and CKD with opposite effect directions. Although Graham et al.[1] report an eGFR association of rs56121637 about 600kB away from this variant, we observed no LD with this variant (r^2=0.093). However, rs56121637 was also genome-wide significant in our analysis (p=8.3x10^{-10}). Sveinbjornsson et al.[17] also found this variant to be associated with creatinine. Both groups proposed *RNF128* as the causal gene.

Conditional analysis revealed that there is another independent variant at this locus, namely rs111410539 not in LD with the variants mentioned above (r^2<0.1). Due to the small effect allele frequency of the variants, the respective credible set were large comprising 408 respectively 1583 variants. Of note, rs181497961 did not receive the highest PP of its credible set, which was attributed to rs111775083 with a higher effect allele frequency of 5.6%. No eQTL colocalizations were detected for this locus. The index variant is in the gene-body of *MORC4* and *CLDN2*. Although, several high CADD-score variants within gene bodies are in the CS (**Supplementary Figure 7**), we consider the gene *CLDN2* as a highly plausible candidate due to its known role in nephrolithiasis development according to OMIM-ID 300520 and the kidney phenotypes of *CLDN2* knock-out mouse models[18].

Locus 10 (Xq23): The top-SNP of this locus, rs5942852, is best associated for eGFR (overall analysis) showing no sex-interaction. The variant was associated with CKD but not BUN. No correlated GWAS hits were found classifying this association as novel finding. The credible set comprises 54 variants. The top-variant is near RPS5P7 and there is also a high CADD variant nearby (CADD=12.6). However, this gene has no known functional relationship to kidney traits. Although 120kB away, the locus co-localizes with an eQTL of *ACSL4* with the same effect direction in blood (PP(H4)=98%, **Figure 5**) and other tissues. *ACSL4* also known as *FACL4* could be a plausible candidate since it was linked to Alport syndrome[19].

Locus 11 (Xq24): EGFR association was highest in the overall analysis for variant rs16275. The SNP is also associated with BUN and CKD in our study. The variant is in LD with variants reported in Sakaue et al. for association with BUN and eGFR[3] and in Graham et al. for association with eGFR[1]. The credible set contains 139 variants. There are several high CADD variants near or in the genes *SLC25A5* and *SLC25A43* including two missense mutations (*SLC25A5*: Leu111Arg, *SLC25A43*: Pro334Leu, **Supplementary Figure 7**). Moreover, cis-eQTLs of *SLC25A5* co-localize with the eGFR signal with the same effect direction in various tissues including kidney tubulointerstitial tissue (**Figure 5**). Thus, this gene is a highly plausible candidate gene of the locus. Of note, the *SLC25A5* anti-sense 1 long-non-coding RNA is associated with renal cancer prognosis[20].

Locus 12 / 20 (Xq25): The top-variant locus rs5931180 was associated with eGFR overall. The variant was nominally significant for BUN, i.e. kidney-related function is considered likely. The locus overlaps with locus 20 of UA association (top-variant: rs112708523) were we also observed a nominally significant sex-interaction with higher effect sizes in males (**Figure 3**). Of note, in contrast to other overlaps, we here observed the same genetic effect directions for eGFR and UA.

The locus was also found in Sakaue et al.[3] for association with creatinine and UA, but here, we demonstrated sex-interaction of the UA association.

The credible set comprised 66 variants for eGFR and 45 variants for UA with a sharp signal related to the genes *MTND4P24* and *DCAF12L1*. Strong CADD scores near the pseudogene *MTND4P24* were observed. No evidence for eQTL colocalizations were detected. *DCAF12L1* has an ARE 3kb downstream (3'UTR) of the TSS and is higher expressed in males in kidney cortex[13], possibly explaining the sex-differential effect. Therefore, it is considered the likely candidate here.

Locus 13 (Xq26.2): The top-variant rs5933079 is most strongly associated for eGFR in the overall analysis, but the sex-interaction test was significant and the variant was only nominally associated in females. The variant was not associated with BUN but with CKD and UA with opposite effect direction (**Figure 4**). The variant was also detected in Sakaue et al.[3] for association with serum creatinine, but here we suggest sex-differential effects.

The credible set contains 39 variants including variants with strong deleteriousness scores near *FRMD7*, *RAP2C* and within *MST4*, respectively. We observed eQTL colocalizations of *FRMD7*, *RAP2C* and *STK26* in tissues not related to kidney function. *FRMD7* is described as a nystagmus-related gene[21]. An *FRMD7* knock-out mouse model showed no kidney phenotype[22]. There are AREs near *RAP2C* (50kp upstream) and *MST4* (28kB downstream) while both genes are also found to be down-regulated by their AREs[6]. There is additional kidney-related evidence related to *MST4*[23,24]. Moreover, *MST4* was shown to correlate with androgen receptor status in prostate cancer cell lines revealing male-specific functionality[25]. Thus, we propose this gene as the most likely candidate here.

Locus 14 / 21 (Xq26.3): The strongest association at locus 14 was observed for rs5933443 for eGFR (overall analysis) with a significant sex-interaction showing larger effect sizes in females. BUN and UA were also associated with opposite effect direction. The locus overlaps with locus 21 showing UA associations (rs202138804) which is an indel. This locus was also found by Sakaue et al.[3] and Graham

et al.[1] with serum creatinine and eGFR. The credible set of the eGFR index variant comprised 17 variants with high CADD score variants in the gene-body of *PLAC1* (intron modifier). The UA association revealed two independent variants (second independent variant rs7056552). CS of these variants comprised 73 respectively 163 variants. Respective variants are in the gene-bodies of *PLAC1*, *HPRT1*, *FAM122B* and *PHF6*, but the strongest CADD scores were again near *PLAC1*. Three of the genes show AREs in some distance (*PLAC1*, *HPRT1*, *FAM122B*), two also show estrogene response elements (*PLAC1*, *HPRT1*) possibly explaining the sex-interaction. No colocalization signals or LD with eQTLs were observed in kidney relevant tissues. However, *HPRT1* also shows higher expression in females[13]. *HPRT1* encodes hypoxanthine phosphoribosyltransferase, a central enzyme in the generation of purines such as UA. Thus, the biological link to the observed association with UA is closer than the one observed with eGFR. Rare loss-of-function variants in *HPRT1* are a cause of Lesch-Nyhan Syndrome featuring highly elevated levels of UA (OMIM-ID 308000)[26]. In consequence, *HPRT1 is* the most plausible candidate gene at this locus.

Locus 15 / 22 (Xq28): The strongest association with eGFR (overall) was found for chr23:152898260:A:C and with UA (overall) for rs4328011. The signals share the same causal variant because LD between the variants was 1 and PP(H4)=100%. The eGFR top-variant is also associated with BUN with opposite effect direction suggesting relevance for kidney function. The variants were in LD with variants reported by Graham et al. for eGFR[1] and by Sakaue et al. for UA and BUN[3].

The CS of chr23:152898260:A:C contains two variants where the index variant already carried 97% PP. For the UA hit, a second independent variant was detected, namely rs111884516. The respective credible sets comprised 3 respectively 1456 variants. Credible set variants of the top-variants were in proximity to *DUSP9*, which was also reported by Graham et al.[1] and Sakaue et al.[3] *DUSP9* is related to renal cancer[27] but also to diabetes[28]. No eQTL colocalizations in kidney-related tissues was observed. But there is LD to a cis-eQTL of another candidate gene *SLC6A8*, which is a creatine transporter. Moreover, the index variants are also in proximity to *FAM58A* related to STAR syndrome[29]. Thus, the causal gene of this association cannot be considered as clarified.

Locus 17 (Xq13.1): At this locus, we observed an UA-specific association for rs34687188. This association was already observed in Sakaue et al.[3] The credible set contained 146 variants, where the top-variant already carries 93% PP. The SNP is in the gene-body of *PIN4* (intron modifier). The top-variant is also in LD with eQTLs of *PIN4* in non-kidney-related tissues. No eQTL colocalizations were observed. *PIN4* has no known relationship to kidney function. Other nearby genes comprise *CITED1*, *RPS4X* and *HDAC8*. Here, *CITED1* could be a plausible candidate[30].

Supplementary Note 2

**Study**	**Acknowledgement**
ADVANCE	ADVANCE genomic sub-studies were supported by grants from the Ministry of Science and Innovation from the Quebec Government, from Genome Quebec, from the Consortium Québécois du Médicament, from the Canadian Institutes of Health Research and from Medpharmgene, OPTITHERA Inc and Les Laboratoires Servier.
AugUR	Age-related diseases: Understanding Genetic and non-genetic influences - a study at the University of Regensburg. Cohort recruiting and management was funded by the Federal Ministry of Education and Research (BMBF-01ER1206, BMBF-01ER1507, to Iris M. Heid) and by the Deutsche Forschungsgemeinschaft (DFG, German Research Foundation) HE 3690/7-1 to Iris M. Heid. Genome-wide genotyping was funded by the University of Regensburg for the Department of Genetic Epidemiology. The computational work supervised by Iris M. Heid was funded by the Deutsche Forschungsgemeinschaft (DFG, German Research Foundation) – Project-ID 387509280, SFB 1350 and Project-ID 509149993, TRR 374 to Iris M. Heid.
BBJ	The BioBank Japan project is supported by the Ministry of Education, Culture, Sports, Sciences and Technology of Japanese government and the Japan Agency for Medical Research and Development.
BES	The Beijing Eye Study was supported by the National Natural Science Foundation of China (#81170890). It has also been partially supported by National Natural Science Foundation of China (#81570835, #82271086), and the Beijing Municipal of Health Reform and Development Project (#2019-4).
CHS	Cardiovascular Health Study: This research was supported by contracts HHSN268201200036C, HHSN268200800007C, HHSN268201800001C, HL105756, N01HC55222, N01HC85079, N01HC85080, N01HC85081, N01HC85082, N01HC85083, N01HC85086, 75N92021D00006, and grants U01HL080295 and U01HL130114 from the National Heart, Lung, and Blood Institute (NHLBI), with additional contribution from the National Institute of Neurological Disorders and Stroke (NINDS). Additional support was provided by R01AG023629 from the National Institute on Aging (NIA). A full list of principal CHS investigators and institutions can be found at CHS-NHLBI.org. A The provision of genotyping data was supported in part by the National Center for Advancing Translational Sciences, CTSI grant UL1TR001881, and the National Institute of Diabetes and Digestive and Kidney Disease Diabetes Research Center (DRC) grant DK063491 to the Southern California Diabetes Endocrinology Research Center. The content is solely the responsibility of the authors and does not necessarily represent the official views of the National Institutes of Health.
CROATIA-Vis	The CROATIA-Vis study was funded by grants from the Medical Research Council (UK), from the Republic of Croatia Ministry of Science, Education and Sports (108-1080315-0302; 216-1080315-0302) and the Croatian Science Foundation (8875); Data analysis was carried out with support from the MRC HGU "QTL in Health and Disease" core programme (U.MC_UU_00007/10). We thank the staff of

several institutions in Croatia that supported the field work, including Zagreb Medical Schools, the Institute for Anthropological Research in Zagreb, the recruitment team from the Croatian Centre for Global Health, University of Split and all the study participants.

CZECH POST-MONICA	The study was supported by research grant 15-27109A provided by the Health Research Agency of the Ministry of Health, Czech Republic; Krka, tovarna zdravil, d.d., Novo mesto, Slovenia; Servier s.r.o., Czech Republic.
DECODE	The study was funded by deCODE Genetics/Amgen inc. We thank the study subjects for their valuable participation and our colleagues, who contributed to data collection, sample handling, and genotyping.
ESTHER	The ESTHER study was funded by the Saarland state Ministry for Social Affairs, Health, Women and Family Affairs (Saarbrücken, Germany), the Baden-Württemberg state Ministry of Science, Research and Arts (Stuttgart, Germany), the Federal Ministry of Education and Research (Berlin, Germany) and the Federal Ministry of Family Affairs, Senior Citizens, Women and Youth (Berlin, Germany).
FINCAVAS	The Finnish Cardiovascular Study (FINCAVAS) has been financially supported by the Competitive Research Funding of the Tampere University Hospital (Grant 9M048 and 9N035), the Finnish Cultural Foundation, the Finnish Foundation for Cardiovascular Research, the Emil Aaltonen Foundation, Finland, the Tampere Tuberculosis Foundation, EU Horizon 2020 (grant 755320 for TAXINOMISIS; grant 848146 for To_Aition), and the Academy of Finland grant 322098. The authors thank the staff of the Department of Clinical Physiology for collecting the exercise test data.
Finrisk	We thank all individuals who participated in Finrisk surveys. Veikko Salomaa and Mikko Kuokkanen were supported by the Finnish Foundation for Cardiovascular Research. Mikko Kuokkanen was also supported by Päivikki and Sakari Sohlberg Foundation, The Paulo Foundation and Yrjö Jahnsson Foundation. We thank the CSC – IT center for science, Finland, for providing computational resources.
GS:SFHS	Generation Scotland received core support from the Chief Scientist Office of the Scottish Government Health Directorates [CZD/16/6] and the Scottish Funding Council [HR03006] and is currently supported by the Wellcome Trust [216767/Z/19/Z]. Genotyping of the GS:SFHS samples was carried out by the Genetics Core Laboratory at the Edinburgh Clinical Research Facility, University of Edinburgh, Scotland and was funded by the Medical Research Council UK and the Wellcome Trust (Wellcome Trust Strategic Award “STratifying Resilience and Depression Longitudinally” (STRADL) Reference 104036/Z/14/Z) .CH was supported by an MRC Human Genetics Unit programme grant ‘Quantitative traits in health and disease’ (U. MC_UU_00007/10).We are grateful to all the families who took part, the general practitioners and the Scottish School of Primary Care for their help in recruiting them, and the whole Generation Scotland team, which includes interviewers, computer and laboratory technicians, clerical workers, research scientists, volunteers, managers, receptionists, healthcare assistants and nurses.

HCHS/SOL	The Hispanic Community Health Study/Study of Latinos is a collaborative study supported by contracts from the National Heart, Lung, and Blood Institute (NHLBI) to the University of North Carolina (HHSN268201300001I /N01-HC-65233), University of Miami (HHSN268201300004I / N01-HC-65234), Albert Einstein College of Medicine (HHSN268201300002I / N01-HC-65235), University of Illinois at Chicago (HHSN268201300003I / N01-HC-65236 Northwestern Univ), and San Diego State University (HHSN268201300005I / N01-HC-65237). The following Institutes/Centers/Offices have contributed to the HCHS/SOL through a transfer of funds to the NHLBI: National Institute on Minority Health and Health Disparities, National Institute on Deafness and Other Communication Disorders, National Institute of Dental and Craniofacial Research, National Institute of Diabetes and Digestive and Kidney Diseases, National Institute of Neurological Disorders and Stroke, NIH Institution-Office of Dietary Supplements. The Genetic Analysis Center at the University of Washington was supported by NHLBI and NIDCR contracts (HHSN268201300005C AM03 and MOD03). This project was supported by NIH R01 DK117445 and R01 MD012765 (to NF).
HUNT	The Trøndelag Health Study (The HUNT Study) is a collaboration between HUNT Research Centre (Faculty of Medicine and Health Sciences, NTNU, Norwegian University of Science and Technology), Trøndelag County Council, Central Norway Regional Health Authority, and the Norwegian Institute of Public Health.
JHS	The Jackson Heart Study (JHS) is supported and conducted in collaboration with Jackson State University (HHSN268201800013I), Tougaloo College (HHSN268201800014I), the Mississippi State Department of Health (HHSN268201800015I/HHSN26800001) and the University of Mississippi Medical Center (HHSN268201800010I, HHSN268201800011I and HHSN268201800012I) contracts from the National Heart, Lung, and Blood Institute (NHLBI) and the National Institute for Minority Health and Health Disparities (NIMHD). The authors also wish to thank the staffs and participants of the JHS. The views expressed in this manuscript are those of the authors and do not necessarily represent the views of the National Heart, Lung, and Blood Institute; the National Institutes of Health; or the U.S. Department of Health and Human Services. Laura M. Raffield is supported by T32 HL129982 and the National Center for Advancing translational Sciences, National Institutes of Health, through Grant KL2TR002490.
LIFE-Adult, LIFE-Heart, LIFE-Child	LIFE-Adult, LIFE-Heart, and LIFE-Child, are funded by the Leipzig Research Center for Civilization Diseases (LIFE). LIFE is an organizational unit affiliated to the Medical Faculty of the University of Leipzig. LIFE is funded by means of the European Union, by the European Regional Development Fund (ERDF) and by funds of the Free State of Saxony within the framework of the excellence initiative. K.Horn was financed by the German Federal Ministry of Education and Research (BMBF) within the framework of the e:Med research and funding concept (SYMPATH, grant # 01ZX1906B). J.Pott was financed by a grant from the Deutsche Forschungsgemeinschaft (DFG, German Research Foundation), Project number 209933838 - Collaborative Research Center SFB1052 “Obesity Mechanisms” (SFB-1052/4 B11).

Living-biobank	The Multi-Ethnic Cohort Phase 1 (MEC1) and Singapore Health Study 2012 (SH2012) studies are supported by the Singapore National Medical Research Council (NMRC), the Singapore Biomedical Research Council (BMRC) and infrastructure funding from the Singapore Ministry of Health (Population Health Metrics Population Health Metrics and Analytics PHMA), National University of Singapore and National University Health System, Singapore. We thank all participants, study team and investigators for their contributions to research. We thank all participants, study team and investigators for their contributions to research.<br>Genome Institute of Singapore provided services for genotyping. Genotyping and whole-exome sequencing for Living Biobank was jointly funded the Agency for Science, Technology and Research, Singapore (https://www.a-star.edu.sg/) and Merck Sharp & Dohme Corp., Whitehouse Station, NJ USA (http://www.merck.com).
LURIC	LURIC was supported by the 7th Framework Program of the EU (AtheroRemo, grant agreement number 201668 and RiskyCAD, grant agreement number 305739). LURIC received further support by the H2020 Program TO_AITION (grant agreement number 848146) of the European Union. The work of W.M. and M.E.K. is supported as part of the Competence Cluster of Nutrition and Cardiovascular Health (nutriCARD) which is funded by the German Ministry of Education and Research (grant agreement no. 01EA1411A). The authors thank the LURIC study team involved in patient recruitment as well as sample and data handling, and the laboratory staff at the Ludwigshafen General Hospital and the universities of Freiburg, Ulm and Heidelberg, Germany.
MESA	MESA and the MESA SHARe projects are conducted and supported by the National Heart, Lung, and Blood Institute (NHLBI) in collaboration with MESA investigators. Support for MESA is provided by contracts 75N92020D00001, HHSN268201500003I, N01-HC-95159, 75N92020D00005, N01-HC-95160, 75N92020D00002, N01-HC-95161, 75N92020D00003, N01-HC-95162, 75N92020D00006, N01-HC-95163, 75N92020D00004, N01-HC-95164, 75N92020D00007, N01-HC-95165, N01-HC-95166, N01-HC-95167, N01-HC-95168, N01-HC-95169, UL1-TR-000040, UL1-TR-001079, and UL1-TR-001420, UL1TR001881, DK063491, and R01HL105756. Funding for SHARe genotyping was provided by NHLBI Contract N02-HL-64278. Genotyping was performed at Affymetrix (Santa Clara, California, USA) and the Broad Institute of Harvard and MIT (Boston, Massachusetts, USA) using the Affymetrix Genome-Wide Human SNP Array 6.0. The authors thank the other investigators, the staff, and the participants of the MESA study for their valuable contributions. A full list of participating MESA investigators and institutes can be found at http://www.mesa-nhlbi.org.
MVP	This work was supported by the MVP-VA Award #CX00189 (PI A.M.H), and Adriana M. Hung (A.M.H) was supported by VA Award #CX001897.
NEO	The authors of the NEO study thank all individuals who participated in the Netherlands Epidemiology in Obesity study, all participating general practitioners for inviting eligible participants and all research nurses for collection of the data. We thank the NEO study group, Pat van Beelen, Petra Noordijk and Ingeborg de Jonge for the coordination, lab and data

management of the NEO study. The genotyping in the NEO study was supported by the Centre National de Génotypage (Paris, France), headed by Jean-Francois Deleuze. The NEO study is supported by the participating Departments, the Division and the Board of Directors of the Leiden University Medical Center, and by the Leiden University, Research 28 Profile Area Vascular and Regenerative Medicine. Dennis Mook-Kanamori is supported by Dutch Science Organization (ZonMW-VENI Grant 916.14.023).

NESDA

Funding for the infrastructure of the NESDA study (www.nesda.nl) was obtained from the Netherlands Organization for Scientific Research (Geestkracht program grant 10-000-1002); the Center for Medical Systems Biology (CSMB, NWO Genomics), Biobanking and Biomolecular Resources Research Infrastructure (BBMRI-NL), VU University Medical Center, GGZ inGeest, Leiden University Medical Center, Leiden University, GGZ Rivierduinen, University Medical Center Groningen, University of Groningen, Lentis, GGZ Friesland, GGZ Drenthe, Rob Giel Onderzoekscentrum, National Institutes of Health (NIH, R01D0042157-01A, MH081802, Grand Opportunity grants 1RC2 MH089951 and 1RC2 MH089995). Part of the genotyping and analyses were funded by the Genetic Association Information Network (GAIN) of the Foundation for the National Institutes of Health. Computing was supported by BiG Grid, the Dutch e-Science Grid, which is financially supported by NWO.

ORCADES

The Orkney Complex Disease Study (ORCADES) was supported by the Chief Scientist Office of the Scottish Government (CZB/4/276, CZB/4/710), a Royal Society URF to James F. Wilson, the MRC Human Genetics Unit quinquennial programme "QTL in Health and Disease", Arthritis Research UK and the European Union framework program 6 EUROSPAN project (contract no. LSHG-CT-2006-018947). DNA extractions were performed at the Wellcome Trust Clinical Research Facility in Edinburgh. We would like to acknowledge the invaluable contributions of the research nurses in Orkney, the administrative team in Edinburgh, the people of Orkney, and the data analysts in particular Dr Thibaud Boutin for the genotype imputation to the HRC reference panel.

QIMR adolescent

We acknowledge funding by Australian National Health and Medical Research Council (NHMRC) grants 241944, 339462, 389927, 389875, 389891, 389892, 389938, 442915, 442981, 496739, 552485, 552498 and Australian Research Council grants A7960034, A79906588, A79801419, DP0770096, DP0212016, DP0343921 for building and maintaining the adolescent twin family resource through which samples were collected.

QIMR adult

We acknowledge the contributions of many staff in the Genetic Epidemiology Unit, Queensland Institute of Medical Research, in interviewing study participants, sample processing and DNA extraction, and data management. Funding for aspects of this work was provided by the Australian National Health and Medical Research Council (241944, 339462, 389927, 389875, 389891, 389892, 389938, 442915, 442981, 496739, 552485, 552498), the Australian Research Council (A7960034, A79906588, A79801419, DP0770096, DP0212016, DP0343921), the EU 5th Framework Programme GenomEUtwin Project (QLG2-CT-2002-01254), and the U.S. National Institutes of Health (AA07535, AA10248, AA11998, AA13320, AA13321, AA13326, AA14041, AA17688, DA12854, MH66206). G.W.M. was

	supported by National Health and Medical Research Council (NHMRC) Fellowship Schemes.
Singapore Epidemiology of Eye Diseases (SEED) – SCES, SiMES, SINDI	The Singapore Chinese Eye Study (SCES), Singapore Malay Eye Study (SiMES) and Singapore Indian Eye Study (SINDI) are supported by the National Medical Research Council (NMRC), Singapore (grants 0796/2003, 1176/2008, 1149/2008, STaR/0003/2008, 1249/2010, CG/SERI/2010, CIRG/1371/2013, CIRG/1417/2015, CIRG/1417/2015, CIRG/1488/2018, OFLCG/004/2018), and Biomedical Research Council (BMRC), Singapore (grants 08/1/35/19/550 and 09/1/35/19/616).
SHIP/SHIP-Trend	SHIP is part of the Community Medicine Research net of the University of Greifswald, Germany, which is funded by the Federal Ministry of Education and Research (grants no. 01ZZ9603, 01ZZ0103, and 01ZZ0403), the Ministry of Cultural Affairs as well as the Social Ministry of the Federal State of Mecklenburg-West Pomerania, and the network 'Greifswald Approach to Individualized Medicine (GANI_MED)' funded by the Federal Ministry of Education and Research (grant 03IS2061A). Genome-wide data have been supported by the Federal Ministry of Education and Research (grant no. 03ZIK012) and a joint grant from Siemens Healthineers, Erlangen, Germany and the Federal State of Mecklenburg- West Pomerania. The University of Greifswald is a member of the Caché Campus program of the InterSystems GmbH.
Sorbs	This work was supported by grants from the Deutsche Forschungsgemeinschaft (DFG, German Research Foundation – Projektnummer 209933838 – SFB 1052) and from Deutsches Zentrum für Diabetesforschung (DZD, Grant: 82DZD00601).
SP2	SP2 is supported by individual research and clinical scientist award schemes from the Singapore National Medical Research Council (NMRC) and the Singapore Biomedical Research Council (BMRC) and infrastructure funding from the Singapore Ministry of Health (Population Health Metrics Population Health Metrics and Analytics PHMA), National University of Singapore and National University Health System, Singapore. We thank all participants, study team and investigators for their contributions to research.
TRAILS	This research is part of the TRacking Adolescents' Individual Lives Survey (TRAILS). Participating centers of TRAILS include the University Medical Center and University of Groningen, the University of Utrecht, the Radboud University Medical Center, and the Parnassia Psychiatric Institute, all in the Netherlands. TRAILS has been financially supported by various grants from the Netherlands Organization for Scientific Research NWO (Medical Research Council program grant GB-MW 940-38-011; ZonMW Brainpower grant 100-001-004; ZonMw Risk Behavior and Dependence grants 60-60600-97-118; ZonMw Culture and Health grant 261-98-710; Social Sciences Council medium-sized investment grants GB-MaGW 480-01-006 and GB-MaGW 480-07-001; Social Sciences Council project grants GB-MaGW 452-04-314 and GB-MaGW 452-06-004; ZonMw Longitudinal Cohort Research on Early Detection and Treatment in Mental Health Care grant 636340002; NWO large-sized investment grant 175.010.2003.005; NWO Longitudinal Survey and Panel Funding 481-08-013 and 481-11-001; NWO Vici

016.130.002, 453-16-007/2735, and Vi.C 191.021; NWO Gravitation 024.001.003), the Dutch Ministry of Justice (WODC), the European Science Foundation (EuroSTRESS project FP-006), the European Research Council (ERC-2017-STG-757364 and ERC-CoG-2015-681466), Biobanking and Biomolecular Resources Research Infrastructure BBMRI-NL (CP 32), the Gratama foundation, the Jan Dekker foundation, the participating universities, and Accare Centre for Child and Adolescent Psychiatry. Statistical analyses were carried out on the Genetic Cluster Computer (https://www.geneticcluster.org), which is financially supported by the Netherlands Scientific Organization (NWO 480-05-003) along with a supplement from the Dutch Brain Foundation. We are grateful to everyone who participated in this research or worked on this project to make it possible.

TwinGene

TwinGene is a sub-study of the Swedish Twin Registry which is managed by Karolinska Institutet and receives funding through the Swedish Research Council under the grant no 2017-00641. TwinGene was supported by grants from the Swedish Research Council (M-2005-1112), GenomEUtwin (EU/QLRT-2001-01254; QLG2-CT-2002-01254), NIH DK U01-066134, The Swedish Foundation for Strategic Research (SSF). Heart and Lung foundation no. 20070481. Analyses on kidney function traits were supported by grants from Stockholm County Council, The Swedish Society of Medicine (SLS-412071), the Serafimer Hospital Foundation, Stiftelsen för Njursjuka and Njurfonden (F2019-0003).

UKBB

This research has been conducted using the UK Biobank resource under application number 20272.

VIKING

The Viking Health Study – Shetland (VIKING) was supported by the MRC Human Genetics Unit quinquennial programme grant “QTL in Health and Disease”. DNA extractions and genotyping were performed at the Edinburgh Clinical Research Facility, University of Edinburgh. We acknowledge the contributions of the research nurses in Shetland, the administrative team in Edinburgh, the people of Shetland, and the data analysts, in particular Dr Thibaud Boutin for the genotype imputation to the HRC reference panel.

WGHS

The WGHS is supported by the National Heart, Lung, and Blood Institute (HL043851 and HL080467) and the National Cancer Institute (CA047988 and UM1CA182913), with support for genotyping provided by Amgen.

YFS

The Young Finns Study has been financially supported by the Academy of Finland: grants 322098, 286284, 134309 (Eye), 126925, 121584, 124282, 129378 (Salve), 117797 (Gendi), and 141071 (Skidi); the Social Insurance Institution of Finland; Competitive State Research Financing of the Expert Responsibility area of Kuopio, Tampere and Turku University Hospitals (grant X51001); Juho Vainio Foundation; Paavo Nurmi Foundation; Finnish Foundation for Cardiovascular Research ; Finnish Cultural Foundation; The Sigrid Juselius Foundation; Tampere Tuberculosis Foundation; Emil Aaltonen Foundation; Yrjö Jahnsson Foundation; Signe and Ane Gyllenberg Foundation; Diabetes Research Foundation of Finnish Diabetes Association; EU Horizon 2020 (grant 755320 for TAXINOMISIS and grant 848146 for To Aition); European Research Council (grant 742927 for MULTIEPIGEN

project); Tampere University Hospital Supporting Foundation, Finnish Society of Clinical Chemistry and the Cancer Foundation Finland.
We thank the teams that collected data at all measurement time points; the persons who participated as both children and adults in these longitudinal studies; and biostatisticians Irina Lisinen, Johanna Ikonen, Noora Kartiosuo, Ville Aalto, and Jarno Kankaanranta for data management and statistical advice.

Supplementary Figures

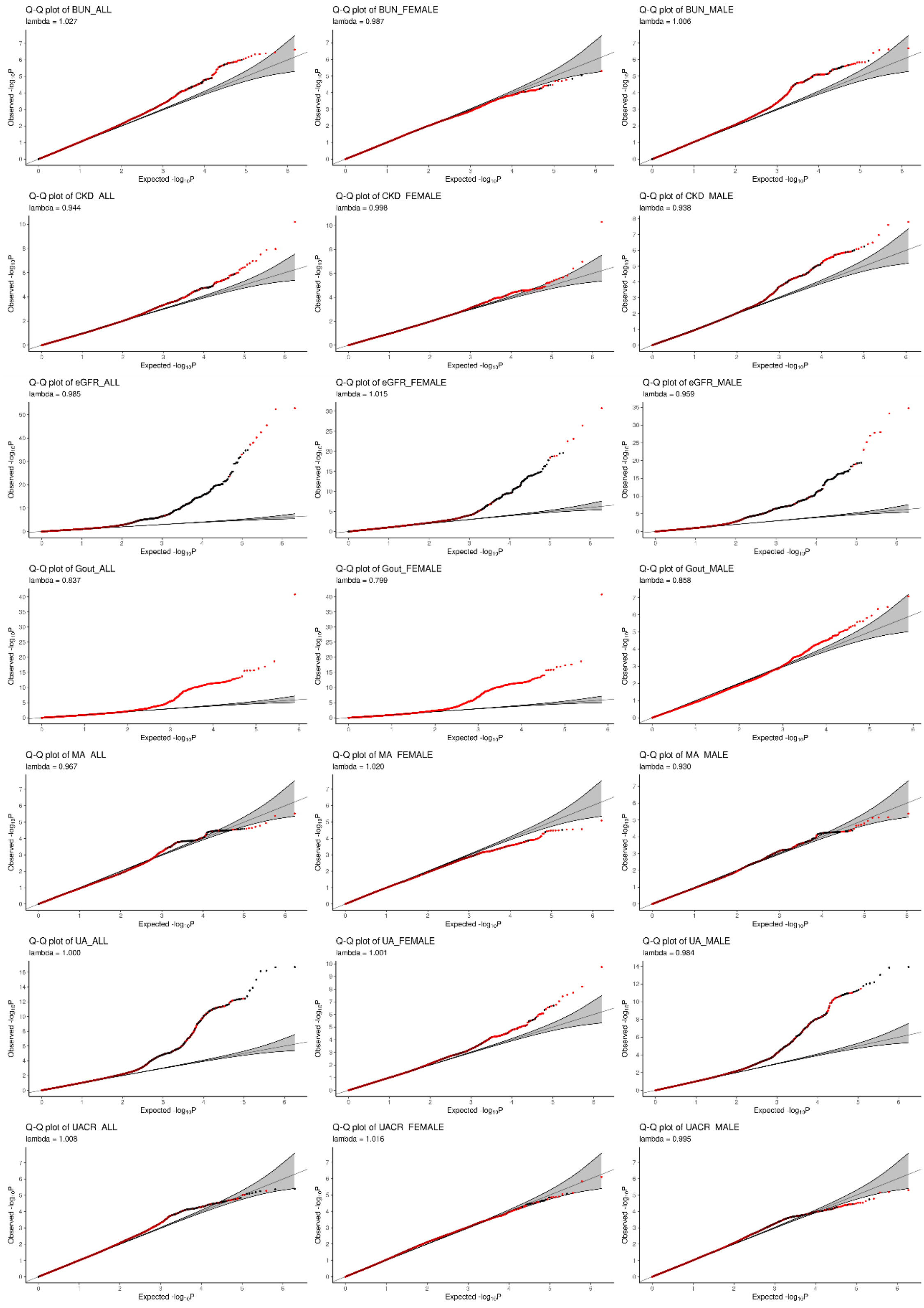

Supplementary Figure 1 (QQ-Plots and Genomic inflation factors): We present quantile-quantile plots and Genomic inflation factors of all traits and groups analysed. SNPs removed during the quality filtering process were displayed in red. For gout all SNPs were discarded due to low numbers of studies and sample size. SNP numbers are provided in **Supplementary Data 3**.

Supplementary Figure 2 (Regional association plots of all identified loci): We present regional association plots of all 22 identified loci for the respective lead traits and for the analysis groups overall, male and female. Note that for locus 7, we have two index variants due to a female-specific signal in addition to the signal in the overall analysis.

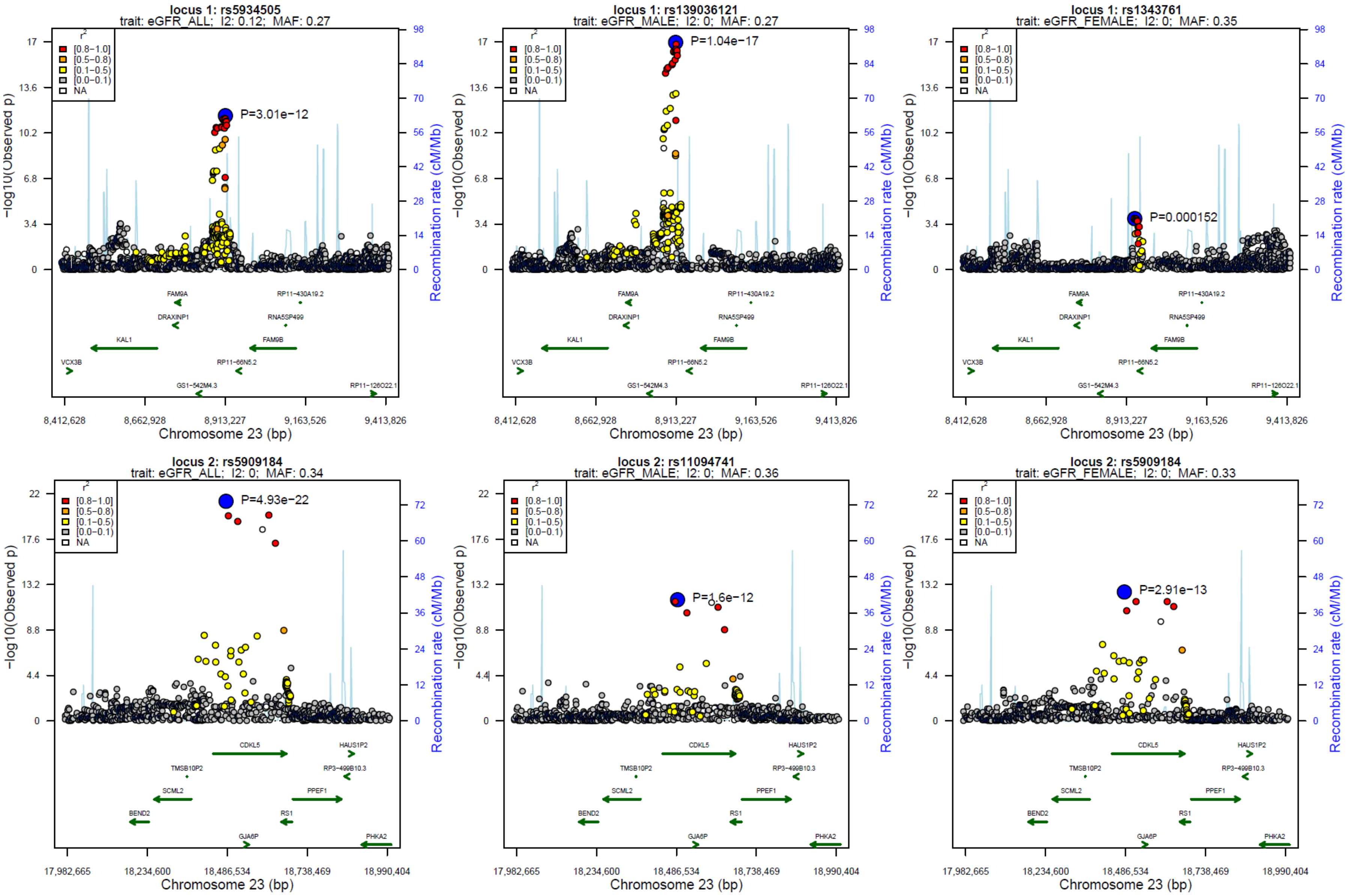

locus 1: rs5934505
trait: eGFR_ALL; I2: 0.12; MAF: 0.27
P=3.01e−12
locus 1: rs139036121
trait: eGFR_MALE; I2: 0; MAF: 0.27
P=1.04e−17
locus 1: rs1343761
trait: eGFR_FEMALE; I2: 0; MAF: 0.35
P=0.000152
locus 2: rs5909184
trait: eGFR_ALL; I2: 0; MAF: 0.34
P=4.93e−22
locus 2: rs11094741
trait: eGFR_MALE; I2: 0; MAF: 0.36
P=1.6e−12
locus 2: rs5909184
trait: eGFR_FEMALE; I2: 0; MAF: 0.33
P=2.91e−13
r2
[0.8−1.0]
[0.5−0.8)
[0.1−0.5)
[0.0−0.1)
NA
−log10(Observed p)
Recombination rate (cM/Mb)
Chromosome 23 (bp)
8,412,628
8,662,928
8,913,227
9,163,526
9,413,826
17,982,665
18,234,600
18,486,534
18,738,469
18,990,404
FAM9A
DRAXINP1
KAL1
VCX3B
GS1−542M4.3
RP11−430A19.2
RNA5SP499
FAM9B
RP11−66N5.2
RP11−126O22.1
CDKL5
TMSB10P2
SCML2
BEND2
GJA6P
HAUS1P2
RP3−499B10.3
PPEF1
RS1
PHKA2

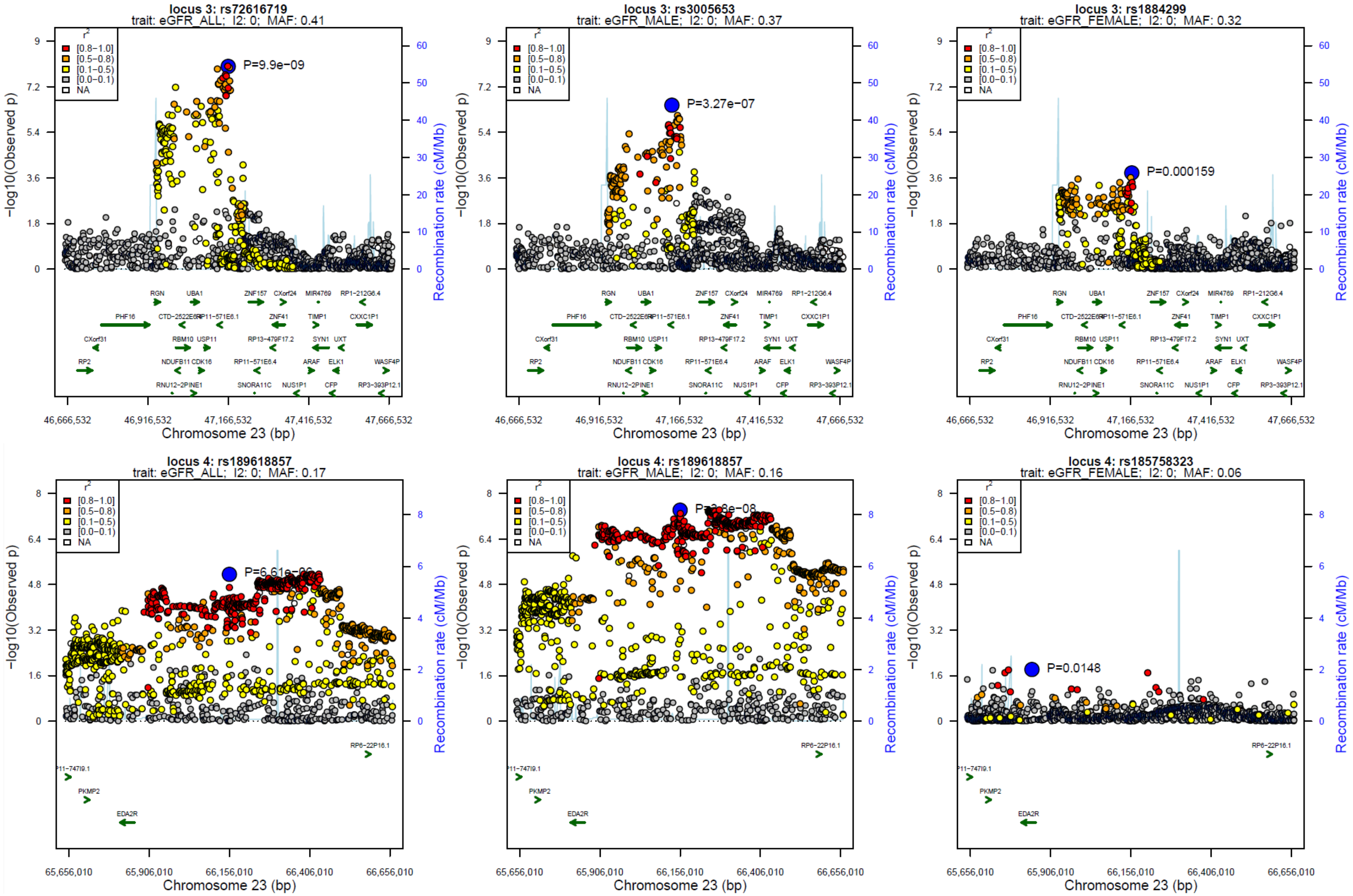
locus 3: rs72616719
trait: eGFR_ALL; I2: 0; MAF: 0.41
P=9.9e-09
locus 3: rs3005653
trait: eGFR_MALE; I2: 0; MAF: 0.37
P=3.27e-07
locus 3: rs1884299
trait: eGFR_FEMALE; I2: 0; MAF: 0.32
P=0.000159
r2
[0.8−1.0]
[0.5−0.8)
[0.1−0.5)
[0.0−0.1)
NA
−log10(Observed p)
Recombination rate (cM/Mb)
Chromosome 23 (bp)
46,666,532
46,916,532
47,166,532
47,416,532
47,666,532
RGN
UBA1
ZNF157
CXorf24
MIR4769
RP1−212G6.4
PHF16
CTD−2522E6.1
RP11−571E6.1
ZNF41
TIMP1
CXXC1P1
CXorf31
RBM10
USP11
RP13−479F17.2
SYN1
UXT
RP2
NDUFB11
CDK16
RP11−571E6.4
ARAF
ELK1
WASF4P
RNU12−2P
INE1
SNORA11C
NUS1P1
CFP
RP3−393P12.1
locus 4: rs189618857
trait: eGFR_ALL; I2: 0; MAF: 0.17
locus 4: rs189618857
trait: eGFR_MALE; I2: 0; MAF: 0.16
locus 4: rs185758323
trait: eGFR_FEMALE; I2: 0; MAF: 0.06
P=0.0148
65,656,010
65,906,010
66,156,010
66,406,010
66,656,010
RP6−22P16.1
PKMP2
EDA2R

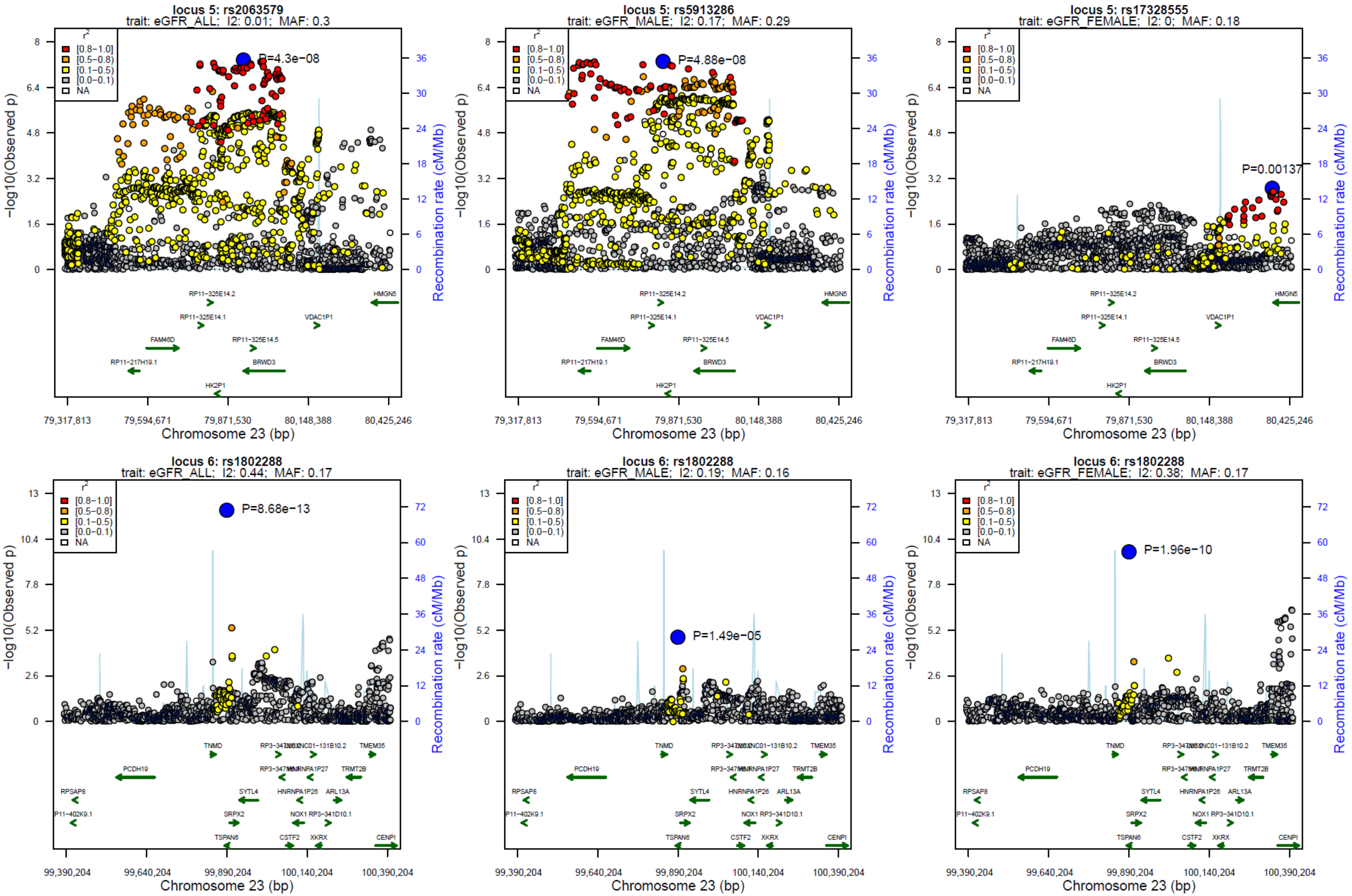
locus 5: rs2063579
trait: eGFR_ALL; I2: 0.01; MAF: 0.3
P=4.3e-08
locus 5: rs5913286
trait: eGFR_MALE; I2: 0.17; MAF: 0.29
P=4.88e-08
locus 5: rs17328555
trait: eGFR_FEMALE; I2: 0; MAF: 0.18
P=0.00137
r2
[0.8−1.0]
[0.5−0.8)
[0.1−0.5)
[0.0−0.1)
NA
−log10(Observed p)
Recombination rate (cM/Mb)
Chromosome 23 (bp)
79,317,813
79,594,671
79,871,530
80,148,388
80,425,246
RP11−325E14.2
RP11−325E14.1
FAM46D
RP11−217H19.1
HK2P1
RP11−325E14.5
BRWD3
VDAC1P1
HMGN5
locus 6: rs1802288
trait: eGFR_ALL; I2: 0.44; MAF: 0.17
P=8.68e−13
locus 6: rs1802288
trait: eGFR_MALE; I2: 0.19; MAF: 0.16
P=1.49e−05
locus 6: rs1802288
trait: eGFR_FEMALE; I2: 0.38; MAF: 0.17
P=1.96e−10
99,390,204
99,640,204
99,890,204
100,140,204
100,390,204
TNMD
PCDH19
RPSAP8
P11−402K9.1
SYTL4
SRPX2
TSPAN6
HNRNPA1P26
NOX1
CSTF2
XKRX
ARL13A
RP3−341D10.1
TRMT2B
TMEM35
CENPI

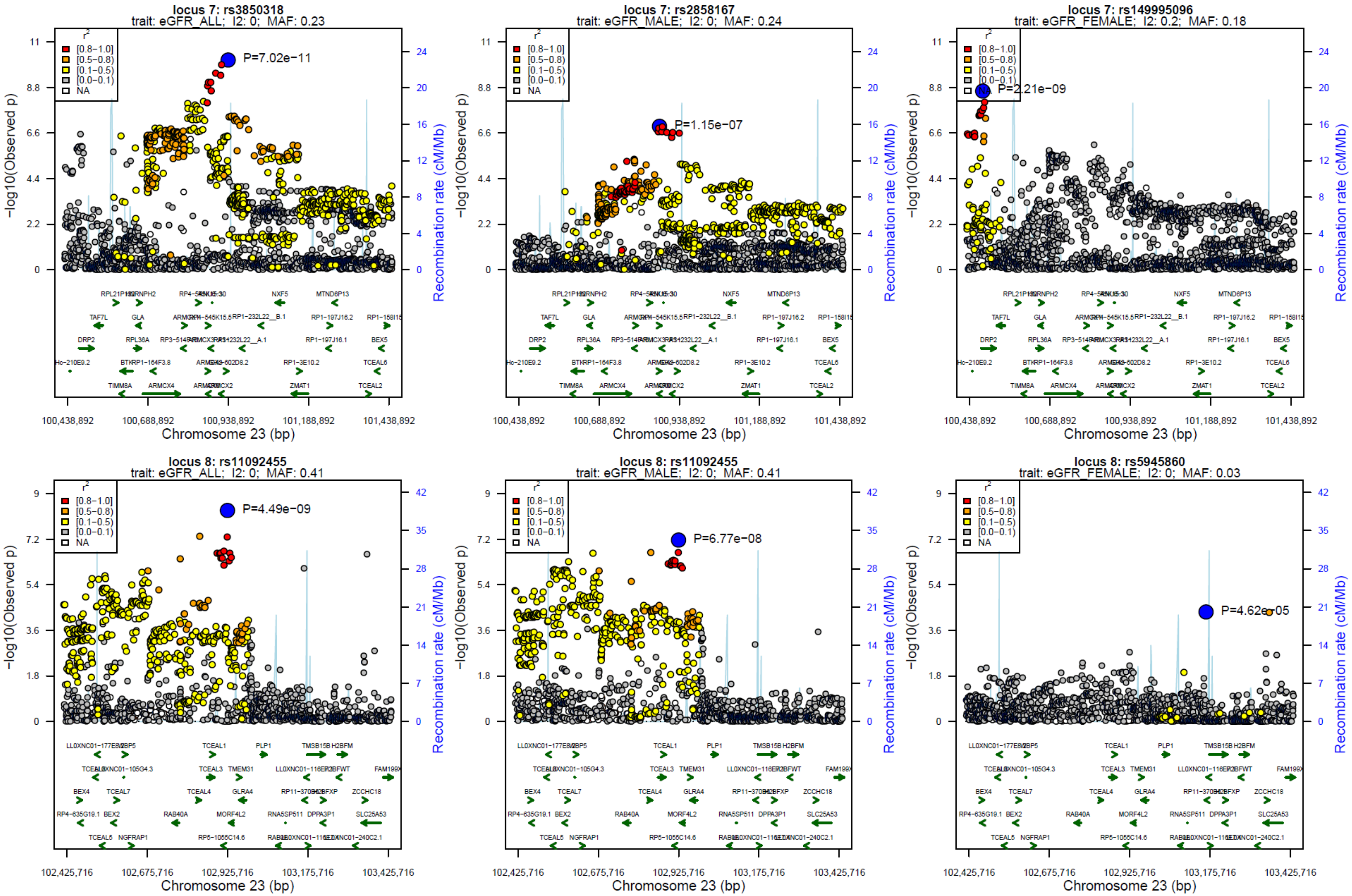
locus 7: rs3850318
trait: eGFR_ALL; I2: 0; MAF: 0.23
P=7.02e−11
locus 7: rs2858167
trait: eGFR_MALE; I2: 0; MAF: 0.24
P=1.15e−07
locus 7: rs149995096
trait: eGFR_FEMALE; I2: 0.2; MAF: 0.18
P=2.21e−09
locus 8: rs11092455
trait: eGFR_ALL; I2: 0; MAF: 0.41
P=4.49e−09
locus 8: rs11092455
trait: eGFR_MALE; I2: 0; MAF: 0.41
P=6.77e−08
locus 8: rs5945860
trait: eGFR_FEMALE; I2: 0; MAF: 0.03
P=4.62e−05
r2
[0.8−1.0]
[0.5−0.8)
[0.1−0.5)
[0.0−0.1)
NA
−log10(Observed p)
Recombination rate (cM/Mb)
Chromosome 23 (bp)
100,438,892
100,688,892
100,938,892
101,188,892
101,438,892
102,425,716
102,675,716
102,925,716
103,175,716
103,425,716

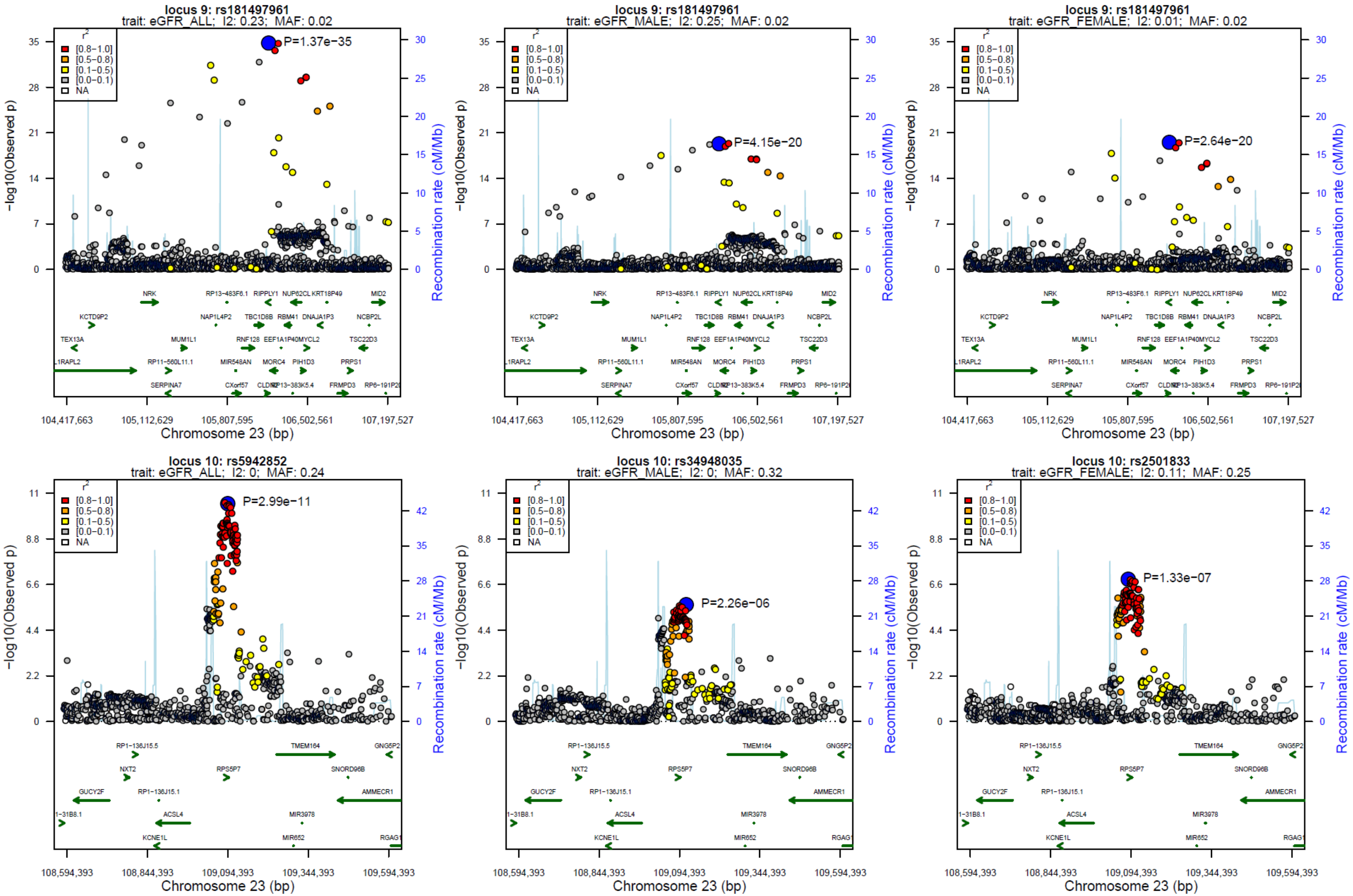

locus 9: rs181497961
trait: eGFR_ALL; I2: 0.23; MAF: 0.02
P=1.37e−35
locus 9: rs181497961
trait: eGFR_MALE; I2: 0.25; MAF: 0.02
P=4.15e−20
locus 9: rs181497961
trait: eGFR_FEMALE; I2: 0.01; MAF: 0.02
P=2.64e−20
locus 10: rs5942852
trait: eGFR_ALL; I2: 0; MAF: 0.24
P=2.99e−11
locus 10: rs34948035
trait: eGFR_MALE; I2: 0; MAF: 0.32
P=2.26e−06
locus 10: rs2501833
trait: eGFR_FEMALE; I2: 0.11; MAF: 0.25
P=1.33e−07
r²
[0.8−1.0]
[0.5−0.8)
[0.1−0.5)
[0.0−0.1)
NA
−log10(Observed p)
Recombination rate (cM/Mb)
Chromosome 23 (bp)
104,417,663
105,112,629
105,807,595
106,502,561
107,197,527
108,594,393
108,844,393
109,094,393
109,344,393
109,594,393

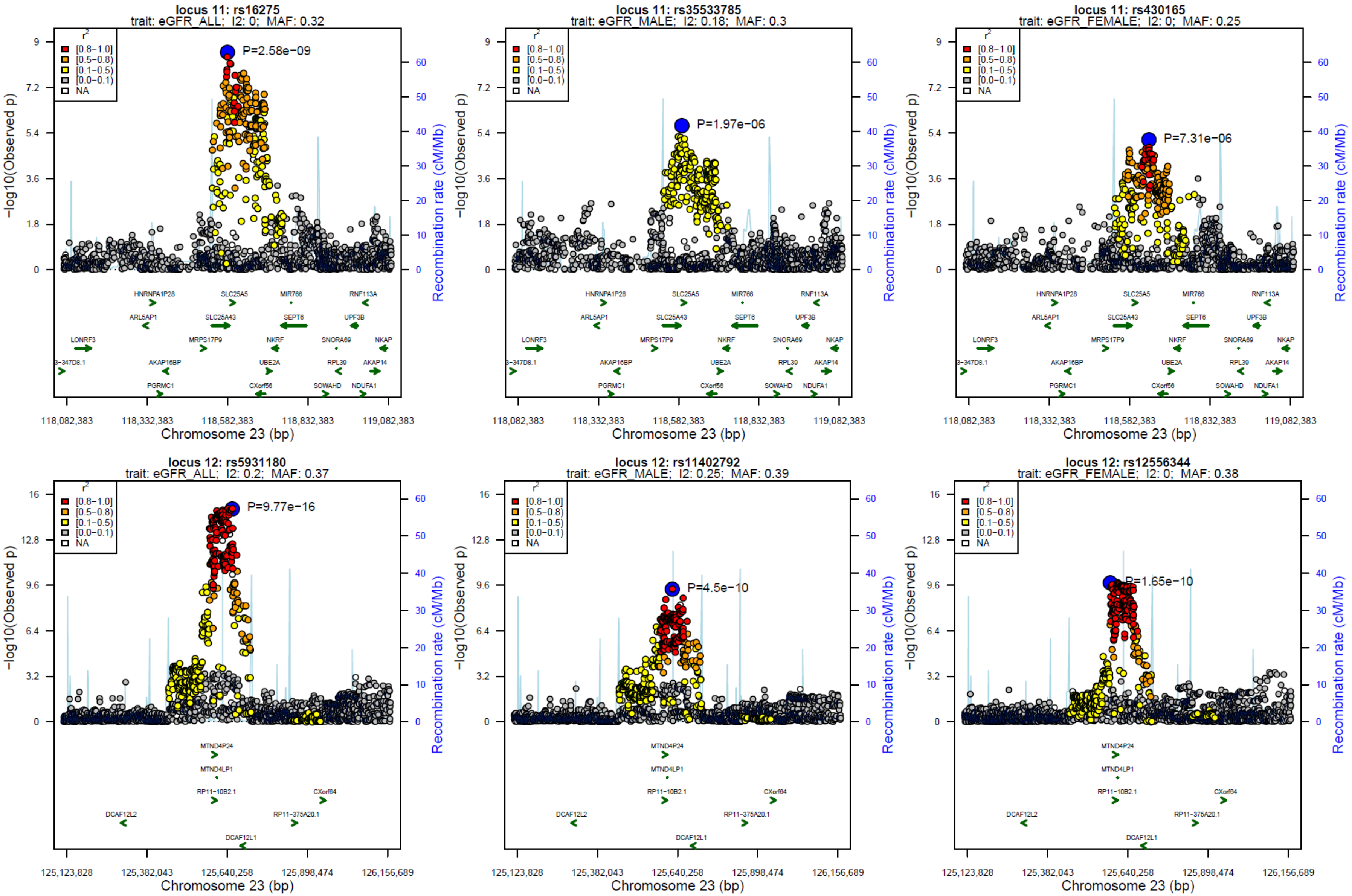

locus 11: rs16275
trait: eGFR_ALL; I2: 0; MAF: 0.32
P=2.58e−09
locus 11: rs35533785
trait: eGFR_MALE; I2: 0.18; MAF: 0.3
P=1.97e−06
locus 11: rs430165
trait: eGFR_FEMALE; I2: 0; MAF: 0.25
P=7.31e−06
locus 12: rs5931180
trait: eGFR_ALL; I2: 0.2; MAF: 0.37
P=9.77e−16
locus 12: rs11402792
trait: eGFR_MALE; I2: 0.25; MAF: 0.39
P=4.5e−10
locus 12: rs12556344
trait: eGFR_FEMALE; I2: 0; MAF: 0.38
P=1.65e−10
[0.8−1.0]
[0.5−0.8)
[0.1−0.5)
[0.0−0.1)
NA
−log10(Observed p)
Recombination rate (cM/Mb)
Chromosome 23 (bp)
118,082,383
118,332,383
118,582,383
118,832,383
119,082,383
125,123,828
125,382,043
125,640,258
125,898,474
126,156,689
HNRNPA1P28
SLC25A5
MIR766
RNF113A
ARL5AP1
SLC25A43
SEPT6
UPF3B
LONRF3
MRPS17P9
NKRF
SNORA69
NKAP
AKAP16BP
UBE2A
RPL39
AKAP14
PGRMC1
CXorf56
SOWAHD
NDUFA1
MTND4P24
MTND4LP1
RP11-10B2.1
CXorf64
DCAF12L2
RP11-375A20.1
DCAF12L1

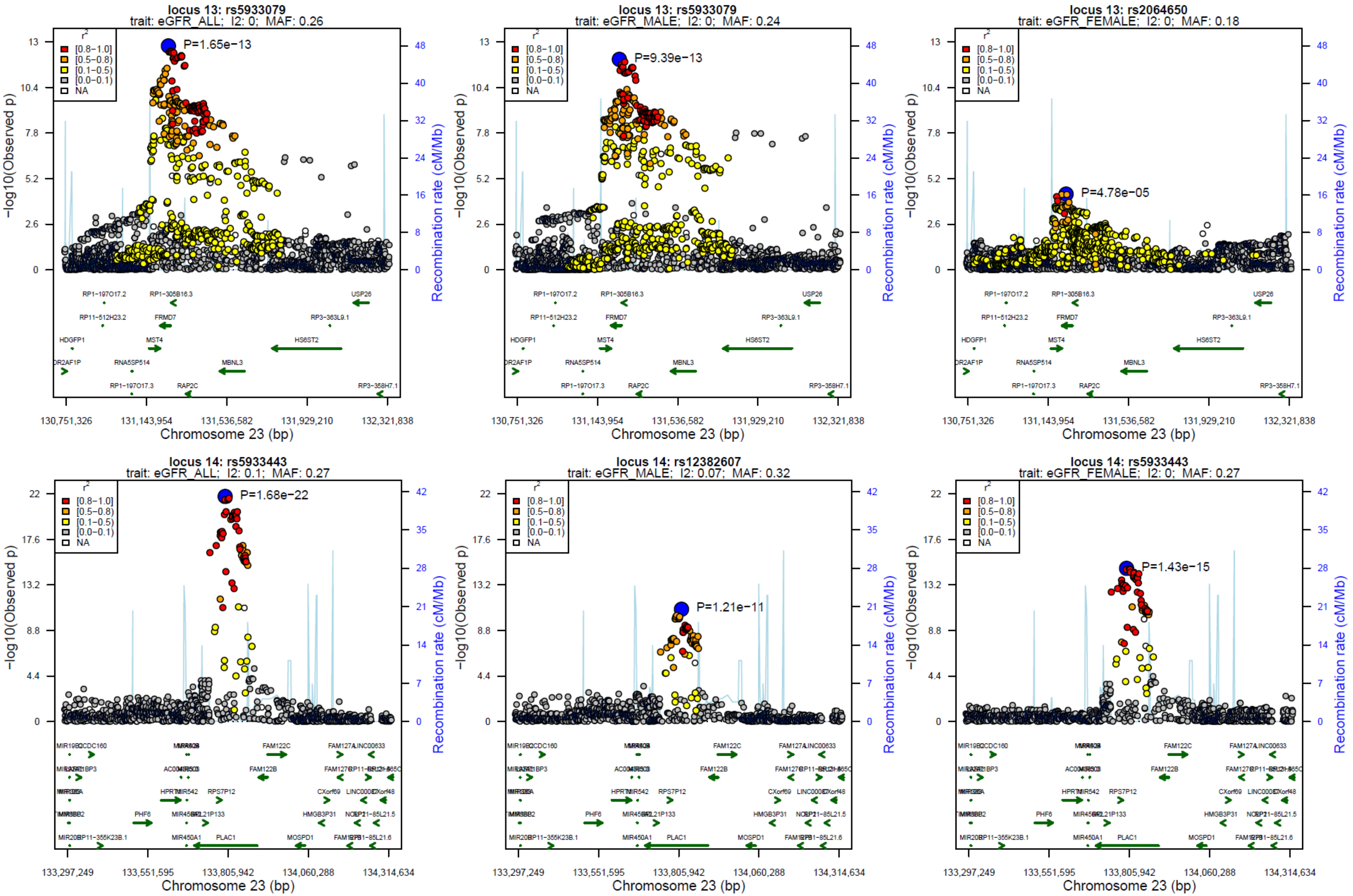
locus 13: rs5933079
trait: eGFR_ALL; I2: 0; MAF: 0.26
P=1.65e−13
locus 13: rs5933079
trait: eGFR_MALE; I2: 0; MAF: 0.24
P=9.39e−13
locus 13: rs2064650
trait: eGFR_FEMALE; I2: 0; MAF: 0.18
P=4.78e−05
locus 14: rs5933443
trait: eGFR_ALL; I2: 0.1; MAF: 0.27
P=1.68e−22
locus 14: rs12382607
trait: eGFR_MALE; I2: 0.07; MAF: 0.32
P=1.21e−11
locus 14: rs5933443
trait: eGFR_FEMALE; I2: 0; MAF: 0.27
P=1.43e−15
r2
[0.8−1.0]
[0.5−0.8)
[0.1−0.5)
[0.0−0.1)
NA
−log10(Observed p)
Recombination rate (cM/Mb)
Chromosome 23 (bp)
130,751,326
131,143,954
131,536,582
131,929,210
132,321,838
133,297,249
133,551,595
133,805,942
134,060,288
134,314,634

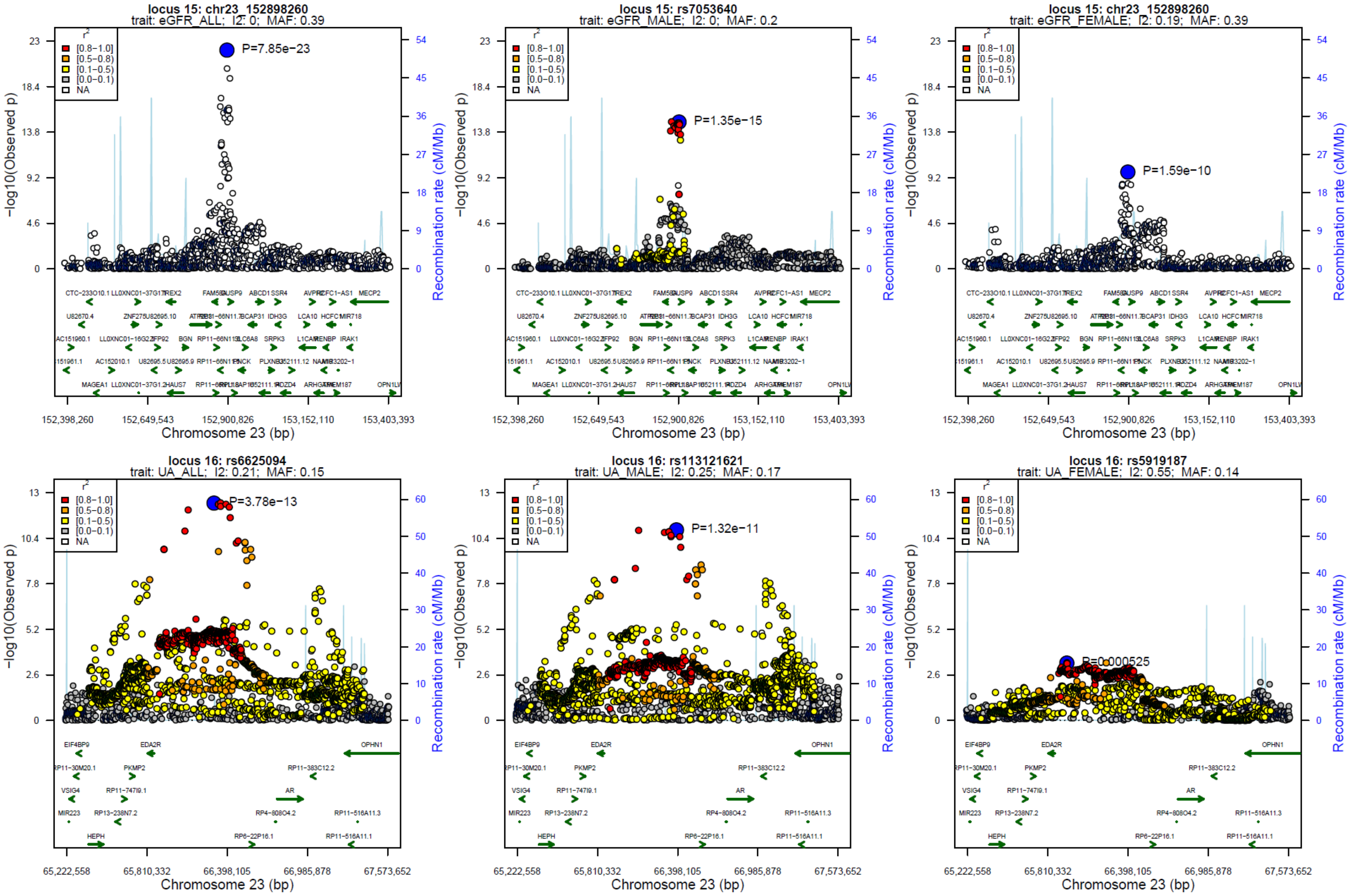

locus 15: chr23_152898260
trait: eGFR_ALL; I2: 0; MAF: 0.39
P=7.85e-23
locus 15: rs7053640
trait: eGFR_MALE; I2: 0; MAF: 0.2
P=1.35e-15
locus 15: chr23_152898260
trait: eGFR_FEMALE; I2: 0.19; MAF: 0.39
P=1.59e-10
locus 16: rs6625094
trait: UA_ALL; I2: 0.21; MAF: 0.15
P=3.78e-13
locus 16: rs113121621
trait: UA_MALE; I2: 0.25; MAF: 0.17
P=1.32e-11
locus 16: rs5919187
trait: UA_FEMALE; I2: 0.55; MAF: 0.14
P=0.000525
r2
[0.8−1.0]
[0.5−0.8)
[0.1−0.5)
[0.0−0.1)
NA
−log10(Observed p)
Recombination rate (cM/Mb)
Chromosome 23 (bp)
152,398,260
152,649,543
152,900,826
153,152,110
153,403,393
65,222,558
65,810,332
66,398,105
66,985,878
67,573,652

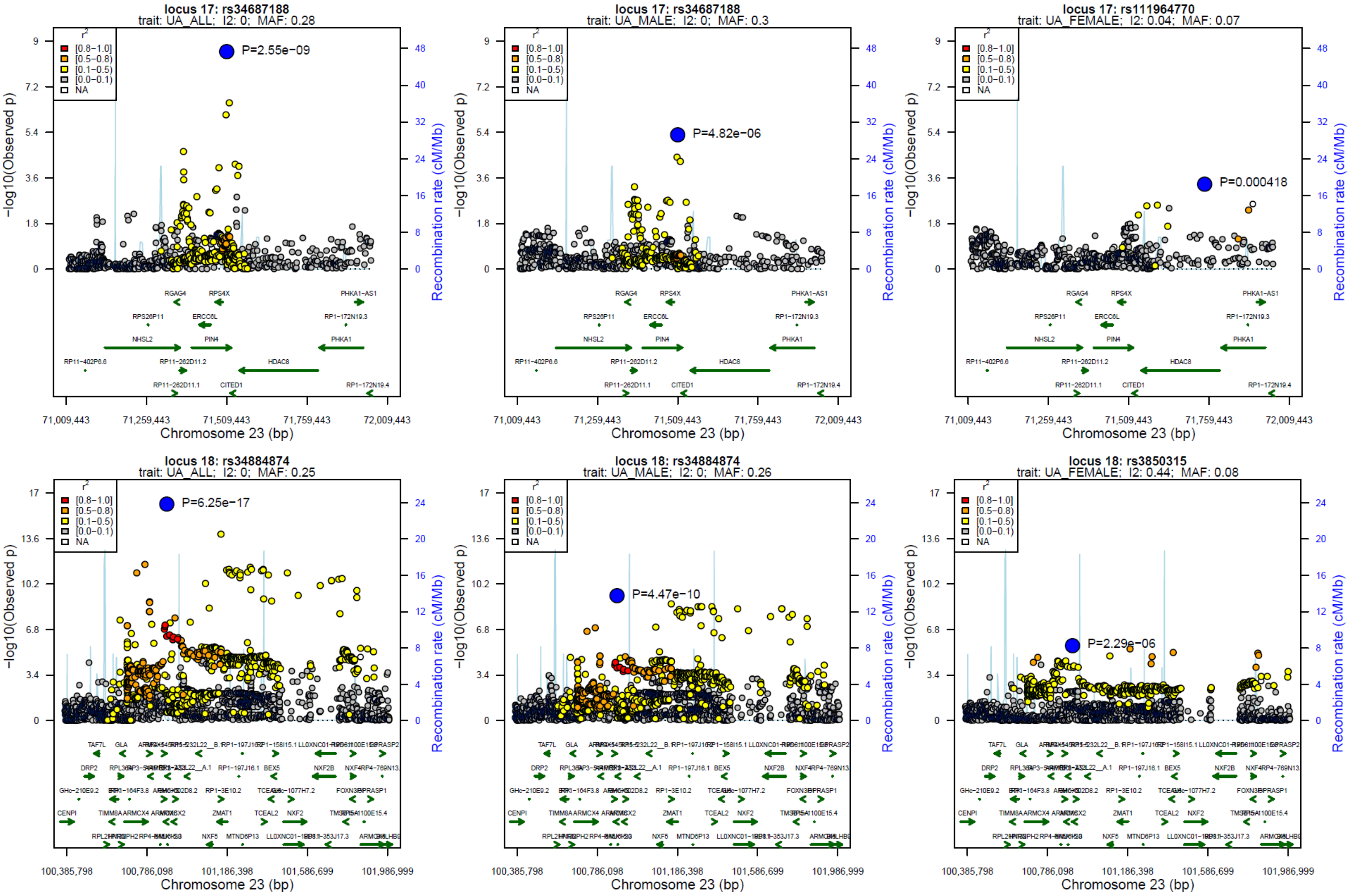
locus 17: rs34687188
trait: UA_ALL; I2: 0; MAF: 0.28
P=2.55e-09
locus 17: rs34687188
trait: UA_MALE; I2: 0; MAF: 0.3
P=4.82e-06
locus 17: rs111964770
trait: UA_FEMALE; I2: 0.04; MAF: 0.07
P=0.000418
locus 18: rs34884874
trait: UA_ALL; I2: 0; MAF: 0.25
P=6.25e-17
locus 18: rs34884874
trait: UA_MALE; I2: 0; MAF: 0.26
P=4.47e-10
locus 18: rs3850315
trait: UA_FEMALE; I2: 0.44; MAF: 0.08
P=2.29e-06
r²
[0.8−1.0]
[0.5−0.8)
[0.1−0.5)
[0.0−0.1)
NA
−log10(Observed p)
Recombination rate (cM/Mb)
Chromosome 23 (bp)
71,009,443
71,259,443
71,509,443
71,759,443
72,009,443
100,385,798
100,786,098
101,186,398
101,586,699
101,986,999
RGAG4
RPS4X
PHKA1−AS1
RPS26P11
ERCC6L
RP1−172N19.3
NHSL2
PIN4
PHKA1
RP11−402P6.6
RP11−262D11.2
HDAC8
RP11−262D11.1
CITED1
RP1−172N19.4
TAF7L
GLA
DRP2
CENPI
ZMAT1
TCEAL2
NXF2
NXF2B
NXF5
MTND6P13
BEX5
FOXN3P
TIMM8A
ARMCX4

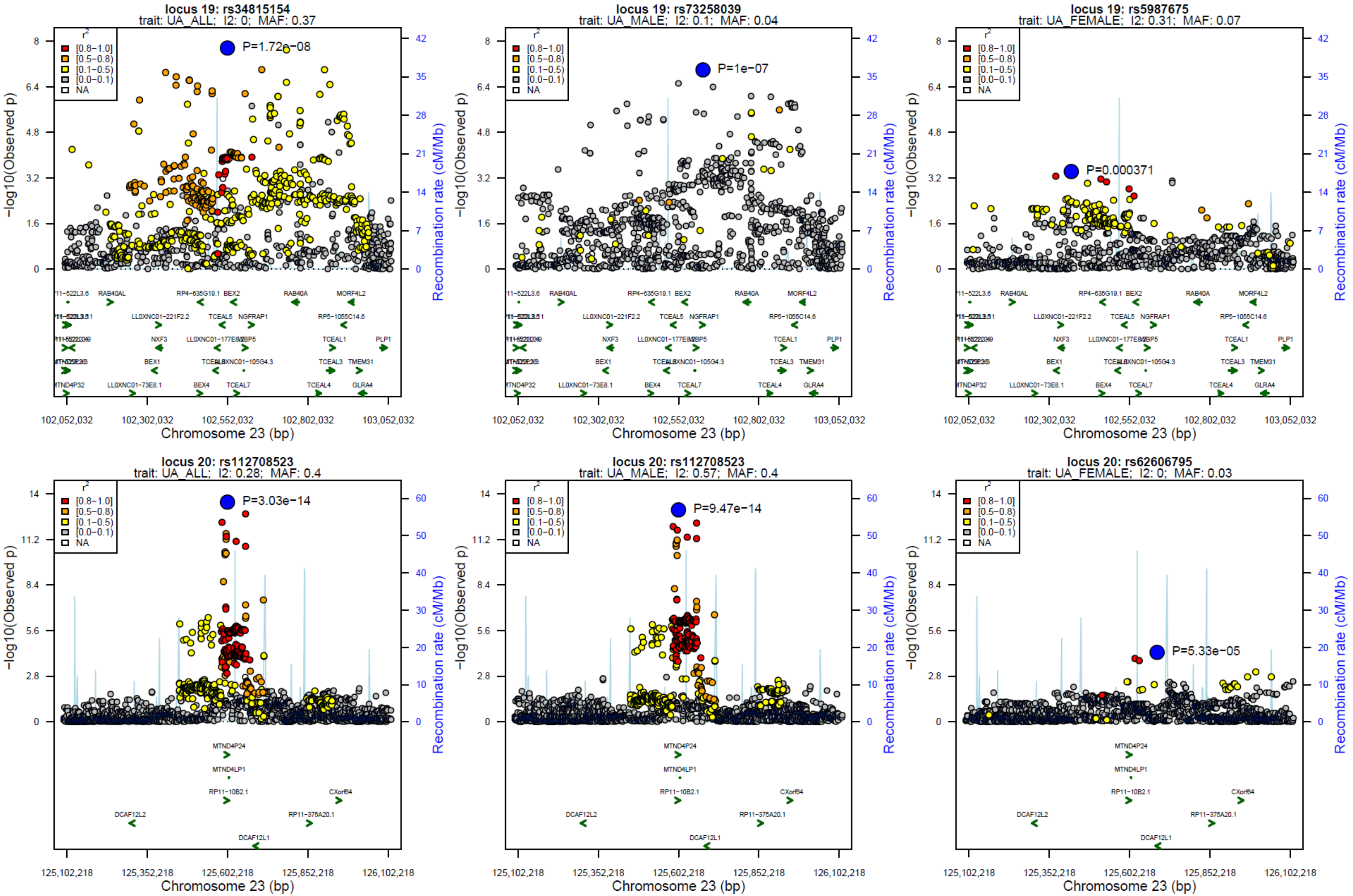
locus 19: rs34815154
trait: UA_ALL; I2: 0; MAF: 0.37
P=1.72e−08
locus 19: rs73258039
trait: UA_MALE; I2: 0.1; MAF: 0.04
P=1e−07
locus 19: rs5987675
trait: UA_FEMALE; I2: 0.31; MAF: 0.07
P=0.000371
locus 20: rs112708523
trait: UA_ALL; I2: 0.28; MAF: 0.4
P=3.03e−14
locus 20: rs112708523
trait: UA_MALE; I2: 0.57; MAF: 0.4
P=9.47e−14
locus 20: rs62606795
trait: UA_FEMALE; I2: 0; MAF: 0.03
P=5.33e−05
r²
[0.8−1.0]
[0.5−0.8)
[0.1−0.5)
[0.0−0.1)
NA
−log10(Observed p)
Recombination rate (cM/Mb)
Chromosome 23 (bp)
102,052,032
102,302,032
102,552,032
102,802,032
103,052,032
125,102,218
125,352,218
125,602,218
125,852,218
126,102,218
RAB40AL
RP4−635G19.1
BEX2
RAB40A
MORF4L2
LL0XNC01−221F2.2
TCEAL5
NGFRAP1
RP5−1055C14.6
NXF3
LL0XNC01−177E8.2
TCEAL1
PLP1
BEX1
TCEAL3
TMEM31
LL0XNC01−73E8.1
BEX4
TCEAL7
TCEAL4
GLRA4
MTND4P24
MTND4LP1
RP11−10B2.1
CXorf64
DCAF12L2
RP11−375A20.1
DCAF12L1

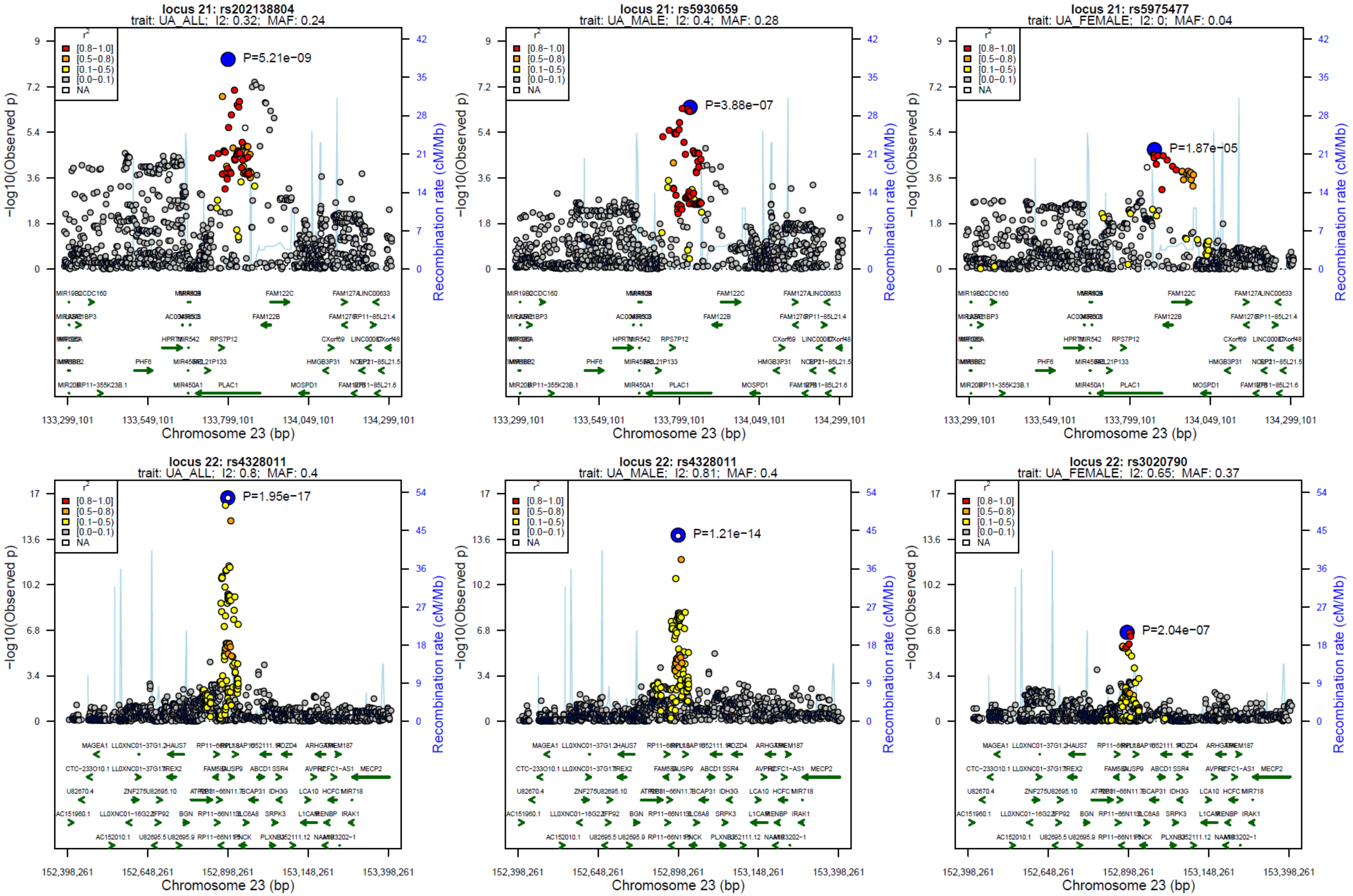
locus 21: rs202138804
trait: UA_ALL; I2: 0.32; MAF: 0.24
P=5.21e-09
locus 21: rs5930659
trait: UA_MALE; I2: 0.4; MAF: 0.28
P=3.88e-07
locus 21: rs5975477
trait: UA_FEMALE; I2: 0; MAF: 0.04
P=1.87e-05
locus 22: rs4328011
trait: UA_ALL; I2: 0.8; MAF: 0.4
P=1.95e-17
locus 22: rs4328011
trait: UA_MALE; I2: 0.81; MAF: 0.4
P=1.21e-14
locus 22: rs3020790
trait: UA_FEMALE; I2: 0.65; MAF: 0.37
P=2.04e-07
r2
[0.8−1.0]
[0.5−0.8)
[0.1−0.5)
[0.0−0.1)
NA
−log10(Observed p)
Recombination rate (cM/Mb)
Chromosome 23 (bp)
133,299,101
133,549,101
133,799,101
134,049,101
134,299,101
152,398,261
152,648,261
152,898,261
153,148,261
153,398,261

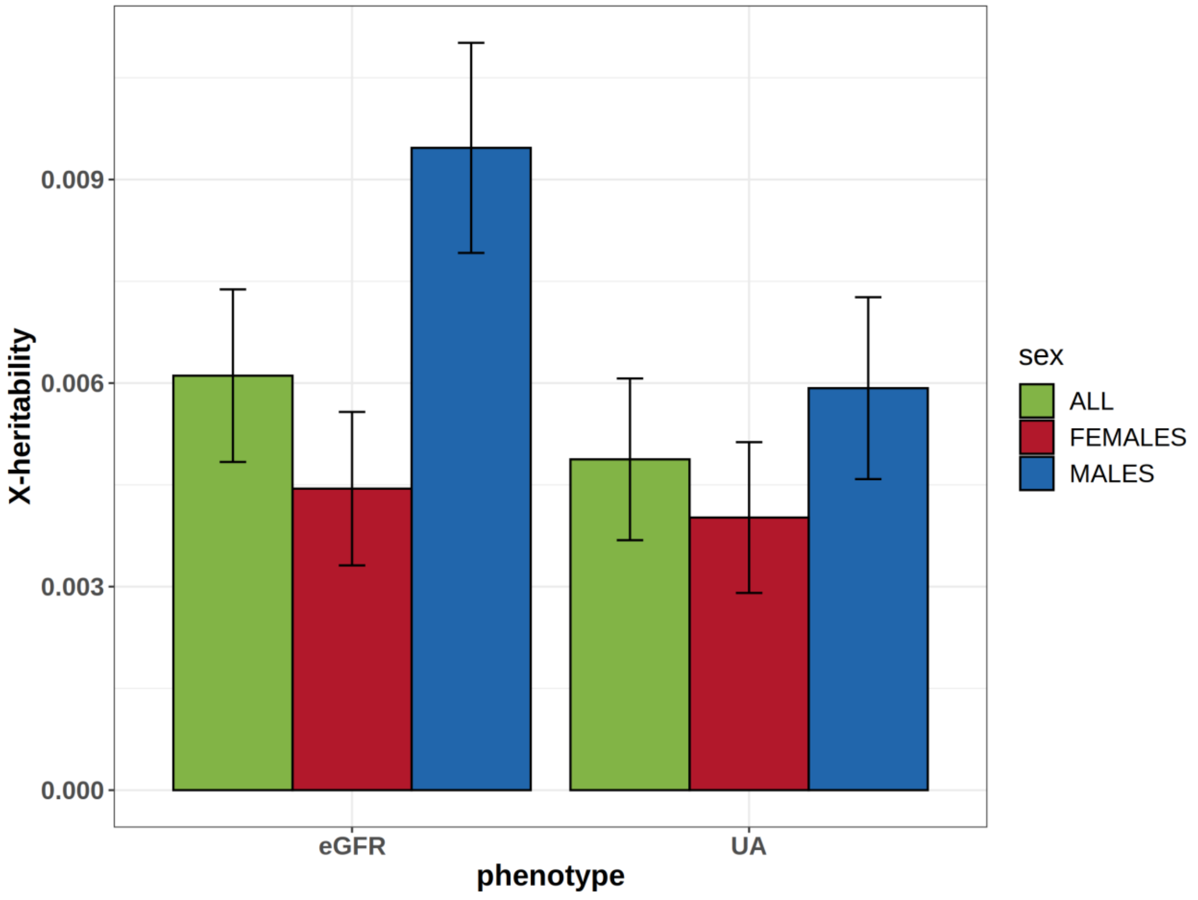

Supplementary Figure 3 (X-chromosomal heritability of eGFR and UA): We provide estimates of X-chromosomal heritability of eGFR and UA based on UKBB for the overall analysis and stratified by sex. Male estimates were significantly larger than the respective estimates for females.

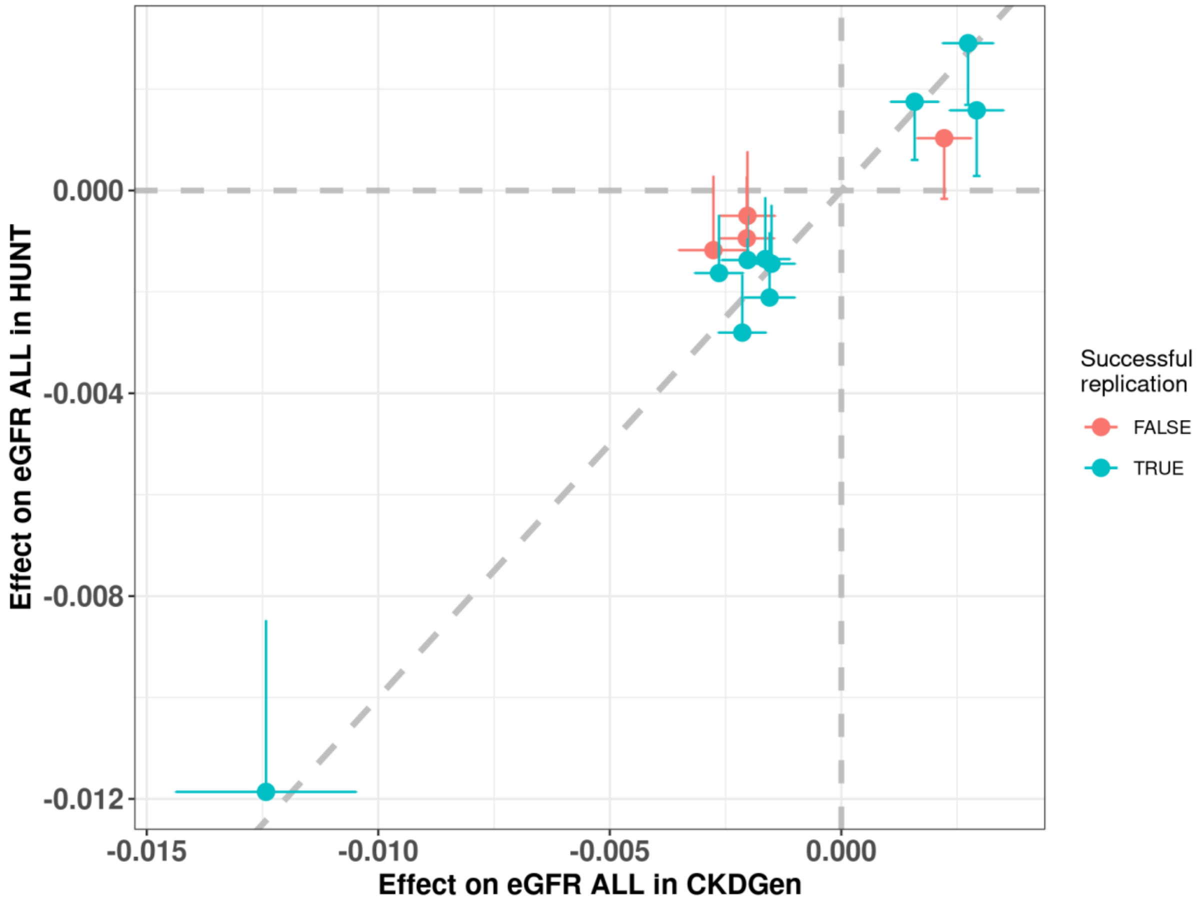

Supplementary Figure 4 (Validation of eGFR hits in HUNT study): The 14 eGFR index variants found in the overall analysis were analyzed in the HUNT study. One-sided nominal significance was assessed and one-side confidence intervals are depicted. Effect directions are consistent throughout and 10 of the 14 variants were significant as expected due to limited power (see also **Supplementary Data 9**).

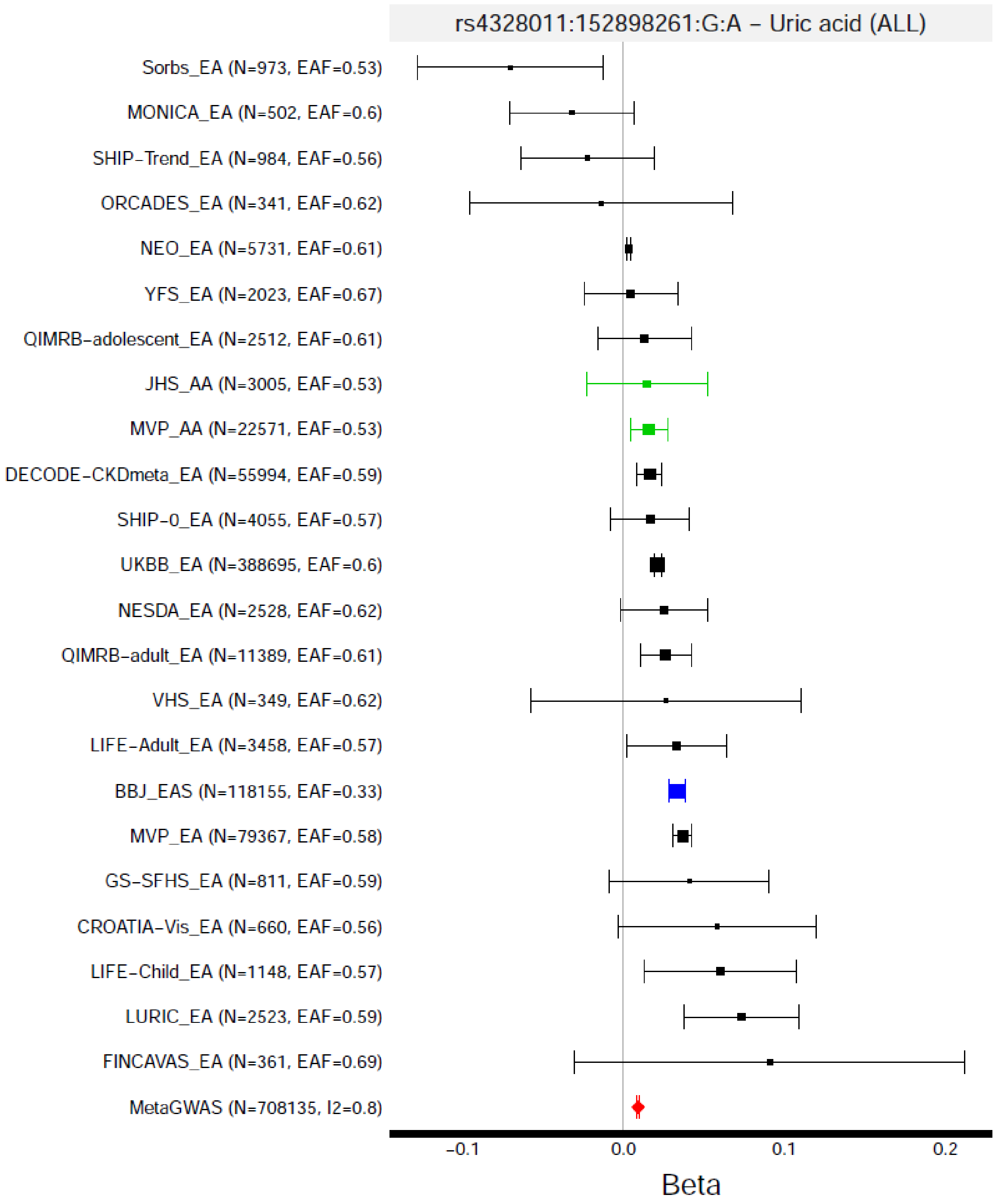

Supplementary Figure 5 (Forest plot of rs4328011): We present the forest plot for variant rs4328011 (locus 22, Xq28) genome-wide significantly associated with uric acid in our meta-analysis ($p=2.0x10^{-17}$). This variant expressed a strong ethnic heterogeneity estimate in MR-MEGA analysis ($p_{Het-Anc}=5.9x10^{-19}$) possibly due to heterogeneity in allele-frequencies between Europeans (black), Asians (blue) and African Americans (green). No bias of effect sizes was observed between ethnicities and MR-MEGA association test also achieved genome-wide significance ($p=1.2x10^{-32}$, see also **Supplementary Data 11**).

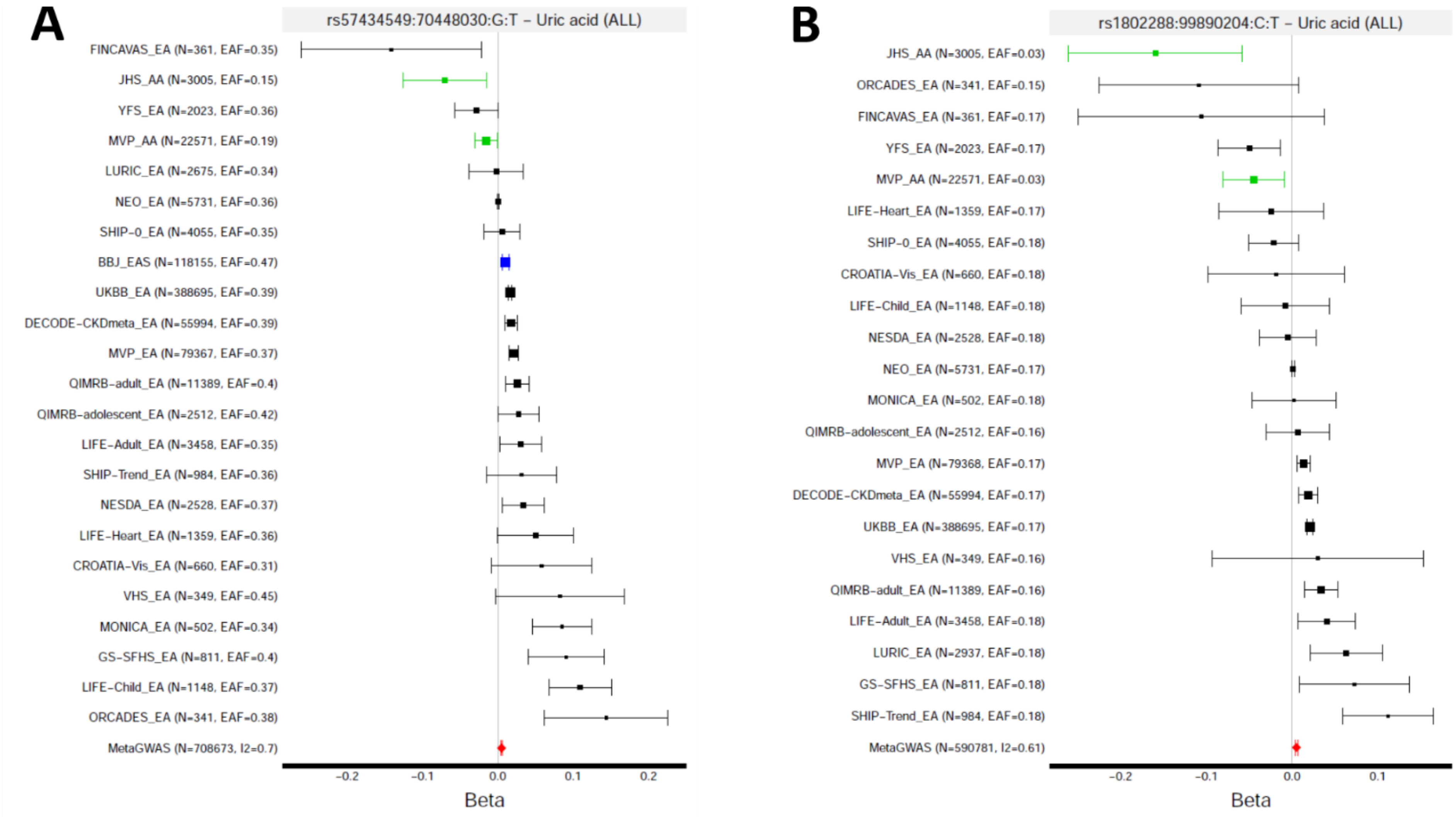

Supplementary Figure 6 (Forest plots of MR-MEGA findings): Two variants not included in loci identified for UA in our trans-ethnic meta-analysis showed genome-wide significance in MR-MEGA analysis of UA. We present the respective forest plots of both variants (sub-figures A and B). Of note, effect sizes tend to be positive in Europeans (black) and Asians (blue) but negative in African Americans (green). See **Supplementary Data 12** for an annotation of these variants.

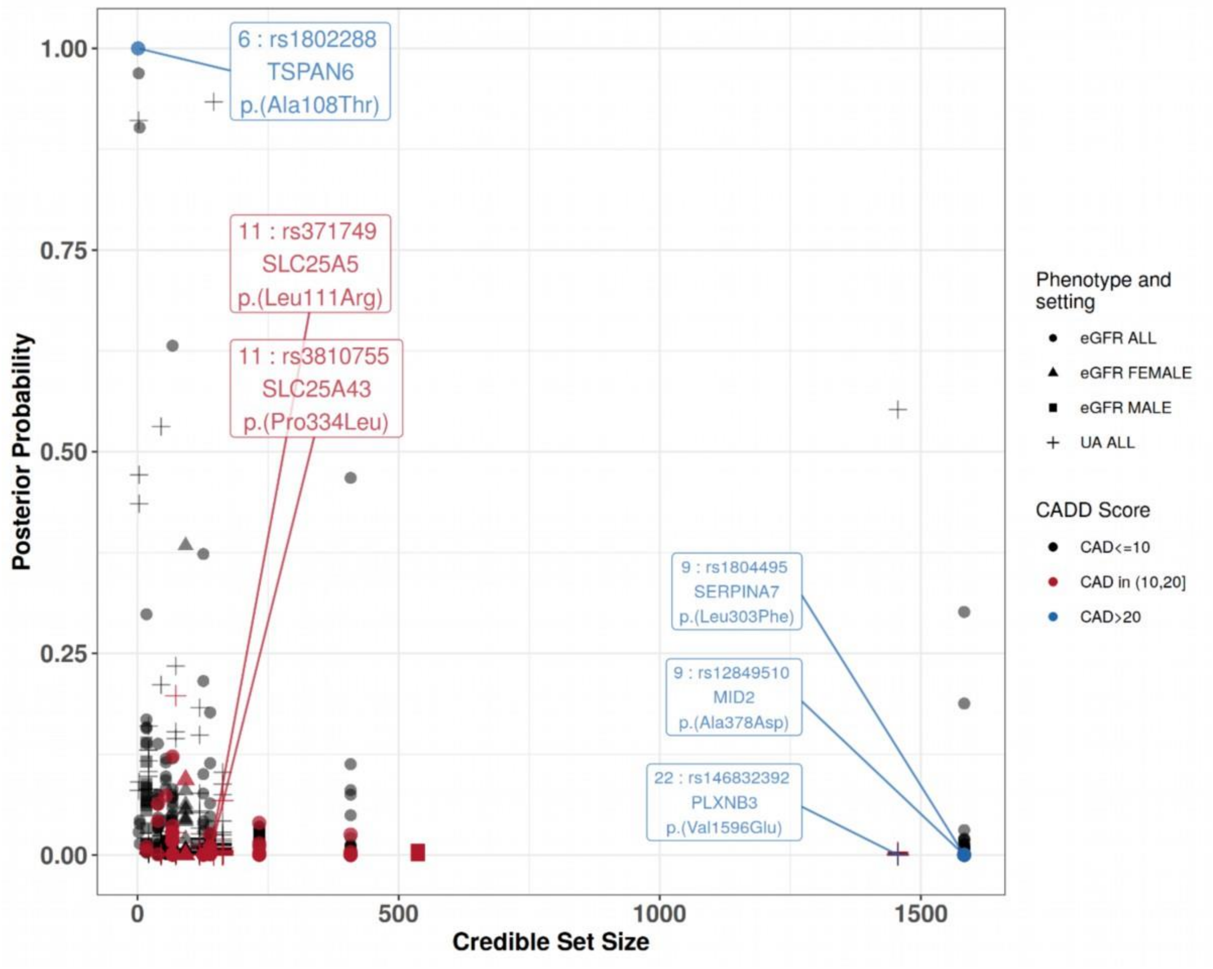

Supplementary Figure 7 (Credible sets and missense mutations): For all index variants (N=23) and their identified secondary independent variants (N=3), we present respective credible set sizes and posterior probabilities of SNPs. Shape of dots correspond to analysis group and phenotype, colour coding corresponds to CADD deleteriousness score. RS-IDs of missense mutations and respective amino-acid exchanges are provided.

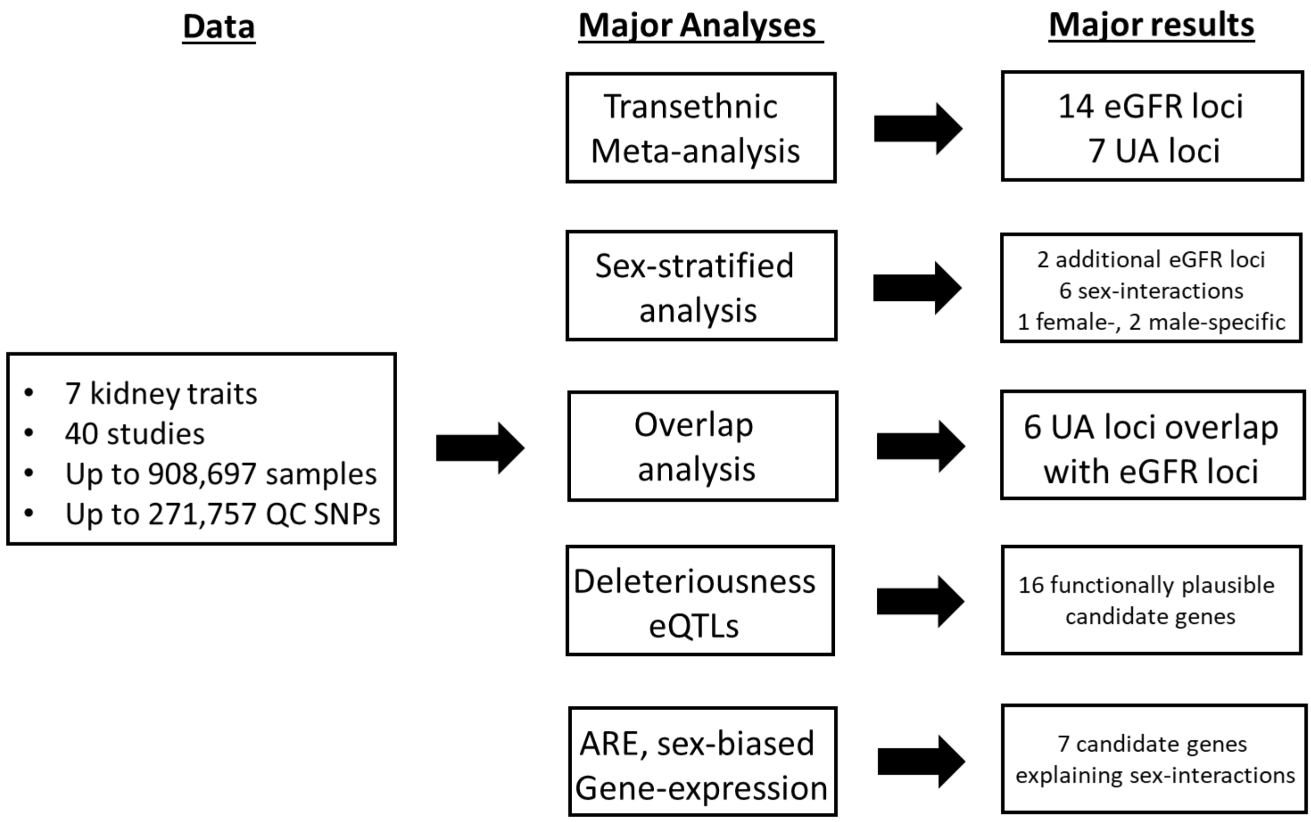

Supplementary Figure 8 (Study design): We present the data basis, major analyses and results of our study.

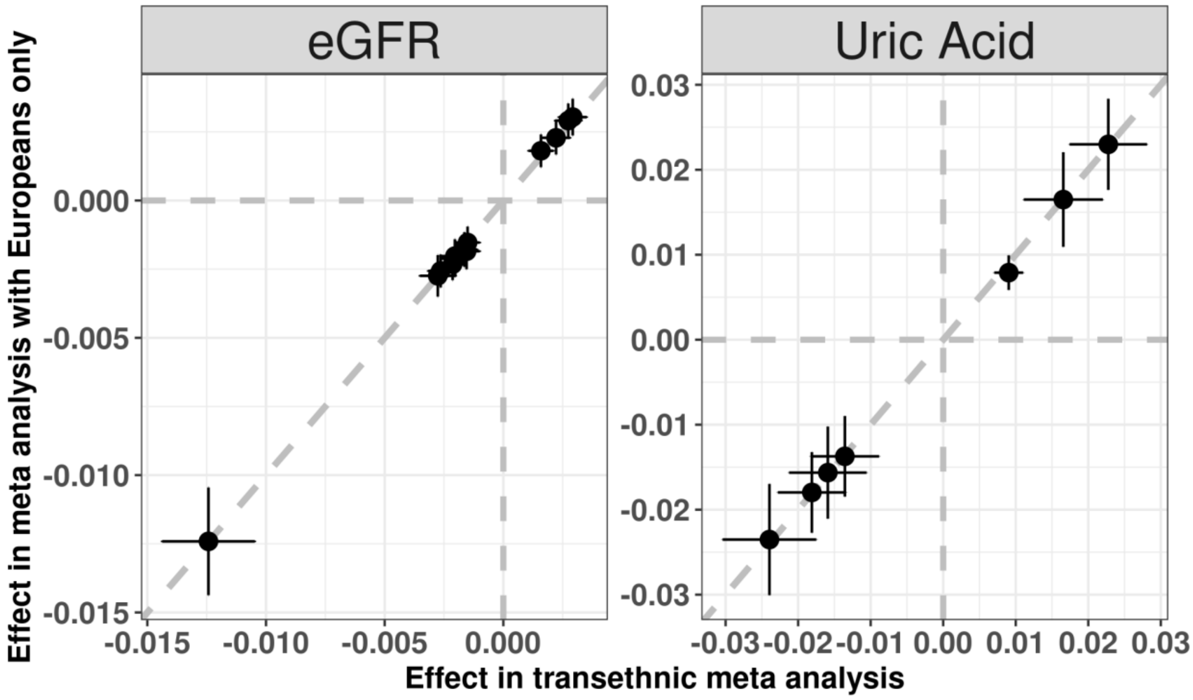

Supplementary Figure 9 (Comparison of effect estimates between trans-ethnic meta-analysis and meta-analysis in Europeans only): Our trans-ethnic meta-analysis is dominated by studies with subjects of European ancestry. Comparing the results of the trans-ethnic meta-analysis with those of the Europeans-only analysis (77.5% of the sample size for eGFR and 79.7% for uric acid) revealed an excellent agreement of effect sizes.

Supplementary References

1. Graham, S. E. *et al.* Sex-specific and pleiotropic effects underlying kidney function identified from GWAS meta-analysis. *Nature communications* **10,** 1847; 10.1038/s41467-019-09861-z (2019).
2. Kanai, M. *et al.* Genetic analysis of quantitative traits in the Japanese population links cell types to complex human diseases. *Nature genetics* **50,** 390–400; 10.1038/s41588-018-0047-6 (2018).
3. Sakaue, S. *et al.* A cross-population atlas of genetic associations for 220 human phenotypes. *Nature genetics* **53,** 1415–1424; 10.1038/s41588-021-00931-x (2021).
4. Fantus, R. J. *et al.* Genetic Susceptibility for Low Testosterone in Men and Its Implications in Biology and Screening: Data from the UK Biobank. *European urology open science* **29,** 36–46; 10.1016/j.euros.2021.04.010 (2021).
5. Yap, C. X. *et al.* Dissection of genetic variation and evidence for pleiotropy in male pattern baldness. *Nature communications* **9,** 5407; 10.1038/s41467-018-07862-y (2018).
6. Wilson, S., Qi, J. & Filipp, F. V. Refinement of the androgen response element based on ChIP-Seq in androgen-insensitive and androgen-responsive prostate cancer cell lines. *Scientific reports* **6,** 32611; 10.1038/srep32611 (2016).
7. Sprovieri, T. *et al.* A novel mutation in the X-linked cyclin-dependent kinase-like 5 (CDKL5) gene associated with a severe Rett phenotype. *American journal of medical genetics. Part A* **149A,** 722–725; 10.1002/ajmg.a.32711 (2009).
8. Si, Y. *et al.* Fisetin decreases TET1 activity and CCNY/CDK16 promoter 5hmC levels to inhibit the proliferation and invasion of renal cancer stem cell. *Journal of cellular and molecular medicine* **23,** 1095–1105; 10.1111/jcmm.14010 (2019).
9. Ni, J.-Y. *et al.* Deubiquitinating enzyme USP11 promotes renal tubular cell senescence and fibrosis via inhibiting the ubiquitin degradation of TGF-β receptor II. *Acta pharmacologica Sinica*; 10.1038/s41401-022-00977-5 (2022).
10. Lan, X. *et al.* EDA2R mediates podocyte injury in high glucose milieu. *Biochimie* **174,** 74–83; 10.1016/j.biochi.2020.04.003 (2020).
11. Bourdeau, V. *et al.* Genome-wide identification of high-affinity estrogen response elements in human and mouse. *Molecular endocrinology (Baltimore, Md.)* **18,** 1411–1427; 10.1210/me.2003-0441 (2004).
12. Hu, H., Zhou, H. & Xu, D. A review of the effects and molecular mechanisms of dimethylcurcumin (ASC-J9) on androgen receptor-related diseases. *Chemical biology & drug design* **97,** 821–835; 10.1111/cbdd.13811 (2021).
13. Oliva, M. *et al.* The impact of sex on gene expression across human tissues. *Science (New York, N.Y.)* **369**; 10.1126/science.aba3066 (2020).
14. Krag, T. O., Gyrd-Hansen, M. & Khurana, T. S. Harnessing the potential of dystrophin-related proteins for ameliorating Duchenne's muscular dystrophy. *Acta physiologica Scandinavica* **171,** 349–358; 10.1046/j.1365-201x.2001.00838.x (2001).
15. Cotton, A. M. *et al.* Analysis of expressed SNPs identifies variable extents of expression from the human inactive X chromosome. *Genome biology* **14,** R122; 10.1186/gb-2013-14-11-r122 (2013).
16. Park, C., Carrel, L. & Makova, K. D. Strong purifying selection at genes escaping X chromosome inactivation. *Molecular biology and evolution* **27,** 2446–2450; 10.1093/molbev/msq143 (2010).

17. Sveinbjornsson, G. *et al.* Rare mutations associating with serum creatinine and chronic kidney disease. *Human molecular genetics* **23,** 6935–6943; 10.1093/hmg/ddu399 (2014).
18. Curry, J. N. *et al.* Claudin-2 deficiency associates with hypercalciuria in mice and human kidney stone disease. *The Journal of clinical investigation* **130,** 1948–1960; 10.1172/JCI127750 (2020).
19. Piccini, M. *et al.* FACL4, a new gene encoding long-chain acyl-CoA synthetase 4, is deleted in a family with Alport syndrome, elliptocytosis, and mental retardation. *Genomics* **47,** 350–358; 10.1006/geno.1997.5104 (1998).
20. Yang, K., Lu, X.-F., Luo, P.-C. & Zhang, J. Identification of Six Potentially Long Noncoding RNAs as Biomarkers Involved Competitive Endogenous RNA in Clear Cell Renal Cell Carcinoma. *BioMed research international* **2018,** 9303486; 10.1155/2018/9303486 (2018).
21. Liu, Z. *et al.* A novel missense mutation in the FERM domain containing 7 (FRMD7) gene causing X-linked idiopathic congenital nystagmus in a Chinese family. *Molecular vision* **19,** 1834–1840 (2013).
22. Salman, A. *et al.* Characterization of the Frmd7 Knock-Out Mice Generated by the EUCOMM/COMP Repository as a Model for Idiopathic Infantile Nystagmus (IIN). *Genes* **11**; 10.3390/genes11101157 (2020).
23. Cansby, E. *et al.* Depletion of protein kinase STK25 ameliorates renal lipotoxicity and protects against diabetic kidney disease. *JCI insight* **5**; 10.1172/jci.insight.140483 (2020).
24. Zeng, X. *et al.* A network-based variable selection approach for identification of modules and biomarker genes associated with end-stage kidney disease. *Nephrology (Carlton, Vic.)* **25,** 775–784; 10.1111/nep.13655 (2020).
25. Sung, V. *et al.* The Ste20 kinase MST4 plays a role in prostate cancer progression. *Cancer research* **63,** 3356–3363 (2003).
26. Jinnah, H. A. *GeneReviews®. HPRT1 Disorders* (Seattle (WA), 1993).
27. Luo, J. *et al.* DUSP9 Suppresses Proliferation and Migration of Clear Cell Renal Cell Carcinoma via the mTOR Pathway. *OncoTargets and therapy* **13,** 1321–1330; 10.2147/OTT.S239407 (2020).
28. Khoubai, F. Z. & Grosset, C. F. DUSP9, a Dual-Specificity Phosphatase with a Key Role in Cell Biology and Human Diseases. *International journal of molecular sciences* **22**; 10.3390/ijms222111538 (2021).
29. Unger, S. *et al.* Mutations in the cyclin family member FAM58A cause an X-linked dominant disorder characterized by syndactyly, telecanthus and anogenital and renal malformations. *Nature genetics* **40,** 287–289; 10.1038/ng.86 (2008).
30. Sparrow, D. B. *et al.* Placental insufficiency associated with loss of Cited1 causes renal medullary dysplasia. *Journal of the American Society of Nephrology : JASN* **20,** 777–786; 10.1681/ASN.2008050547 (2009).
