# Supplementary material for: X-chromosome and kidney function: evidence from a multi-trait genetic analysis of 908,697 individuals reveals sex-specific and sex-differential findings in genes regulated by androgen response elements
Source: Nat Commun. 2024 Jan 18;15:586. doi: 10.1038/s41467-024-44709-1 (PMC10794254; doi:10.1038/s41467-024-44709-1)
Supplement: Supplementary file 3 — Description of Additional Supplementary Files [file 41467_2024_44709_MOESM3_ESM.pdf]

Description of Additional Supplementary Files

File name: Supplementary Data 1
Description: Description of participating studies: study design and phenotype distribution.

File name: Supplementary Data 2
Description: Genotyping and imputation information of participating studies.

File name: Supplementary Data 3
Description: Number of data sets, samples and SNPs contributing to the different association analyses.

File name: Supplementary Data 4
Description: Comparisons between sexes of the 23 index SNPs.

File name: Supplementary Data 5a
Description: Comparisons between phenotypes (look up in all seven kidney traits in the respective top-associated sub-group).

File name: Supplementary Data 5b
Description: Comparisons between phenotypes (look up for all seven kidney traits and all analysis groups).

File name: Supplementary Data 5c
Description: Comparisons between phenotypes (colocalization analyses of physically overlapping loci associated with eGFR respectively UA).

File name: Supplementary Data 6
Description: Independent variants per locus and analysis group.

File name: Supplementary Data 7
Description: Annotation of 99% Credible Sets.

File name: Supplementary Data 8
Description: Co-localization of genetic association signals and eQTLs.

File name: Supplementary Data 9
Description: Validation of eGFR associations in HUNT.

File name: Supplementary Data 10
Description: Look-up of SNPs previously reported for UA, eGFR, creatinine and BUN.

File name: Supplementary Data 11
Description: Results of meta-regression analysis of the 23 index SNPs.

File name: Supplementary Data 12
Description: Additional genome-wide significant associations due to meta-regression.

File name: Supplementary Data 13
Description: Look-up of sex-biased gene-expressions of candidate genes assigned to genetic sex-interactions.

File name: Supplementary Data 14
Description: Co-localization of genetic association signals of CKD traits and testosterone.

File name: Supplementary Data 15
Description: Look-up of eQTL effect directions in case of positive co-localization.
