# Supplementary material for: X-chromosome and kidney function: evidence from a multi-trait genetic analysis of 908,697 individuals reveals sex-specific and sex-differential findings in genes regulated by androgen response elements
Source: Nat Commun. 2024 Jan 18;15:586. doi: 10.1038/s41467-024-44709-1 (PMC10794254; doi:10.1038/s41467-024-44709-1)
Supplement: Supplementary file 5 — Reporting Summary [file 41467_2024_44709_MOESM5_ESM.pdf]

Corresponding author(s): Markus Scholz
Last updated by author(s): Nov 27, 2023

Reporting Summary

Nature Portfolio wishes to improve the reproducibility of the work that we publish. This form provides structure for consistency and transparency in reporting. For further information on Nature Portfolio policies, see our Editorial Policies and the Editorial Policy Checklist.

Statistics

For all statistical analyses, confirm that the following items are present in the figure legend, table legend, main text, or Methods section.

n/a	Confirmed	
☐	☒	The exact sample size (*n*) for each experimental group/condition, given as a discrete number and unit of measurement
☐	☒	A statement on whether measurements were taken from distinct samples or whether the same sample was measured repeatedly
☐	☒	The statistical test(s) used AND whether they are one- or two-sided<br>*Only common tests should be described solely by name; describe more complex techniques in the Methods section.*
☐	☒	A description of all covariates tested
☐	☒	A description of any assumptions or corrections, such as tests of normality and adjustment for multiple comparisons
☐	☒	A full description of the statistical parameters including central tendency (e.g. means) or other basic estimates (e.g. regression coefficient) AND variation (e.g. standard deviation) or associated estimates of uncertainty (e.g. confidence intervals)
☐	☒	For null hypothesis testing, the test statistic (e.g. *F*, *t*, *r*) with confidence intervals, effect sizes, degrees of freedom and *P* value noted<br>*Give P values as exact values whenever suitable.*
☐	☒	For Bayesian analysis, information on the choice of priors and Markov chain Monte Carlo settings
☒	☐	For hierarchical and complex designs, identification of the appropriate level for tests and full reporting of outcomes
☐	☒	Estimates of effect sizes (e.g. Cohen's *d*, Pearson's *r*), indicating how they were calculated

Our web collection on statistics for biologists contains articles on many of the points above.

Software and code

Policy information about availability of computer code

Data collection	R v4.2.2
Data analysis	R v4.2.2, Plink 2.0, GCTA v1.94.0, MR-Mega v0.1.2, Analysis scripts are provided at GitHub https://github.com/GenStatLeipzig/CKDGen_ChrX.

For manuscripts utilizing custom algorithms or software that are central to the research but not yet described in published literature, software must be made available to editors and reviewers. We strongly encourage code deposition in a community repository (e.g. GitHub). See the Nature Portfolio guidelines for submitting code & software for further information.

Data

Policy information about availability of data

All manuscripts must include a data availability statement. This statement should provide the following information, where applicable:
- Accession codes, unique identifiers, or web links for publicly available datasets
- A description of any restrictions on data availability
- For clinical datasets or third party data, please ensure that the statement adheres to our policy

Summary statistics for this study are publicly available at http://ckdgen.imbi.uni-freiburg.de/pico/datasets/Scholz_2023. Other data sets used in this study include NephQTL(https://nephqtl.org/), GTEx V8 data (https://gtexportal.org/home/protectedDataAccess), the HUNT Study (https://www.ntnu.edu/hunt) and the UK Biobank (https://www.ukbiobank.ac.uk/).

Research involving human participants, their data, or biological material

Policy information about studies with human participants or human data. See also policy information about sex, gender (identity/presentation), and sexual orientation and race, ethnicity and racism.

Reporting on sex and gender	Sex was considered in the study design. We provided sex-specific and sex-combined analysis results. We also performed genetic sex-interaction analyses.
Reporting on race, ethnicity, or other socially relevant groupings	Genetic ancestry of study participants was accounted for by performing stratified analyses according to self-reported ancestries and by performing Meta-regression considering estimated genetic ancestries of participating studies (MR-Mega).
Population characteristics	We conducted overall and sex-stratified fixed-effect meta-analyses of X chromosome-wide association scans of seven kidney-related traits and diseases from 40 mainly population-based study groups totaling up to 908,697 individuals with a mean age of 55.7 years (Tables S1 and S2). Specifically, we analyzed eGFR (n=773,980, mean=91.33 ml/min/1.73m²), uric acid (UA; n=710,704, mean=5.09 mg/dl), urinary albumin-to-creatinine ratio (UACR; n=455,053, mean=9.65 mg/g), blood urea nitrogen (BUN; n=180,748, mean = 15.05 mg/dl), CKD (n=908,697, including 40,785 cases), microalbuminuria (MA; n=517,768, 36,578 cases), and gout (n=195,018, 2412 cases). Sex ratios were roughly balanced for all traits (45-59% female, Table S3). About 80% of study participants were of European ancestry.
Recruitment	Different recruitment modalities of participating studies are provided as short study descriptions or specific publications of the respective cohorts.
Ethics oversight	Ethics statements of single studies were provided in the respective acknowledgements.

Note that full information on the approval of the study protocol must also be provided in the manuscript.

Field-specific reporting

Please select the one below that is the best fit for your research. If you are not sure, read the appropriate sections before making your selection.

☒ Life sciences ☐ Behavioural & social sciences ☐ Ecological, evolutionary & environmental sciences

For a reference copy of the document with all sections, see nature.com/documents/nr-reporting-summary-flat.pdf

Life sciences study design

All studies must disclose on these points even when the disclosure is negative.

Sample size	Due to the hypothesis-free nature of genome-wide association studies, we used the maximum number of available sample sizes of the traits considered in this study. Efforts to collect all available studies are performed in the framework of the CKDGen consortium.
Data exclusions	EGFR of adults was estimated based on serum creatinine using the 2009 CKD-EPI equation, winsorizing at 15 and 200 ml/min/1.73m2. Studies of children or adolescents (age ≤18 years) used a revised formula proposed by Schwartz et al.<br>Subjects with incomplete genotype, covariate or target trait data were not analysed.
Replication	During meta-analysis we evaluated the heterogeneity of effect estimates across studies. The HUNT study (N=69,389) was used for validation of the 14 loci associated with eGFR. Effect directions were consistent for all index variants and effect sizes were in good agreement (Figure S4, Pearson's r=0.96, p=1.1x10-8). Ten loci showed nominally significant effects in the HUNT study in accordance with the expected statistical power (Table S9). The variants explained 0.15% of the eGFR variance in HUNT, a value similar to that found in our meta-analysis.
Randomization	Not applicable. We mostly used data from non-randomized observational studies. Covariate effects were considered during regression analysis.
Blinding	No group comparisons were performed. Blinding was not meaningful in our analysis context.

Reporting for specific materials, systems and methods

We require information from authors about some types of materials, experimental systems and methods used in many studies. Here, indicate whether each material, system or method listed is relevant to your study. If you are not sure if a list item applies to your research, read the appropriate section before selecting a response.

Materials & experimental systems

n/a	Involved in the study
☒	☐ Antibodies
☒	☐ Eukaryotic cell lines
☒	☐ Palaeontology and archaeology
☒	☐ Animals and other organisms
☒	☐ Clinical data
☒	☐ Dual use research of concern
☒	☐ Plants

Methods

n/a	Involved in the study
☒	☐ ChIP-seq
☒	☐ Flow cytometry
☒	☐ MRI-based neuroimaging
